# Organotypic Co-Cultures as a Novel 3D Model for Head and Neck Squamous Cell Carcinoma

**DOI:** 10.3390/cancers12082330

**Published:** 2020-08-18

**Authors:** Luca Engelmann, Julia Thierauf, Natalia Koerich Laureano, Hans-Juergen Stark, Elena-Sophie Prigge, Dominik Horn, Kolja Freier, Niels Grabe, Chao Rong, Philippe Federspil, Karim Zaoui, Peter K. Plinkert, Nicole Rotter, Magnus von Knebel Doeberitz, Jochen Hess, Annette Affolter

**Affiliations:** 1 Department of Otorhinolaryngology, Head and Neck Surgery, Experimental Head and Neck Oncology, Heidelberg University Hospital, Im Neuenheimer Feld 400, 69120 Heidelberg, Germany; engelmann.luca@yahoo.de (L.E.); jthierauf@mgh.harvard.edu (J.T.); nataliakoerich@hotmail.com (N.K.L.); rongchaochina@163.com (C.R.); federspil@med.uni-heidelberg.de (P.F.); karim.zaoui@med.uni-heidelberg.de (K.Z.); peter.plinkert@med.uni-heidelberg.de (P.K.P.); j.hess@dkfz-heidelberg.de (J.H.); 2 Department of Pathology, Massachusetts General Hospital, 55 Fruit Street, Boston, MA 02114, USA; 3 Oral Pathology, Federal University of Rio Grande do Sul, Av. Paulo Gama 110, Porto Alegre 90040-060, Brazil; 4 Molecular Mechanisms of Head and Neck Tumors, German Cancer Research Center, Im Neuenheimer Feld 280, 69120 Heidelberg, Germany; 5 Department of Applied Tumor Biology, Institute of Pathology, University of Heidelberg, Im Neuenheimer Feld 224, 69120 Heidelberg, Germany; hj.stark@dkfz-heidelberg.de (H.-J.S.); elena.prigge@med.uni-heidelberg.de (E.-S.P.); magnus.knebel-doeberitz@med.uni-heidelberg.de (M.v.K.D.); 6 Clinical Cooperation Unit Applied Tumor Biology, German Cancer Research Centre, Im Neuenheimer Feld 280, 69120 Heidelberg, Germany; 7 Department of Oral and Maxillofacial Surgery, Saarland University Medical Center, Kirrberger Strasse, 66424 Homburg, Germany; dominik.horn@uks.eu (D.H.); kolja.freier@uks.eu (K.F.); 8 National Center for Tumor Diseases, Hamamatsu TIGA Center, Bioquant, Heidelberg University, Im Neuenheimer Feld 267, 69120 Heidelberg, Germany; niels.grabe@bioquant.uni-heidelberg.de; 9 Department of Pathology, School of Biology & Basic Medical Sciences, Soochow University, 199 Ren-Ai Road, Suzhou Industrial Park, Suzhou 215123, China; 10 Department of Otorhinolaryngology, Head and Neck Surgery, University Hospital Mannheim, Medical Faculty Mannheim of Heidelberg University, Theodor-Kutzer-Ufer 1-3, 68167 Mannheim, Germany; Nicole.Rotter@umm.de

**Keywords:** HNSCC, 3D organotypic co-culture model, invasion, HPV

## Abstract

*Background*: Head and neck squamous cell carcinomas (HNSCC) are phenotypically and molecularly heterogeneous and frequently develop therapy resistance. Reliable patient-derived 3D tumor models are urgently needed to further study the complex pathogenesis of these tumors and to overcome treatment failure. *Methods*: We developed a three-dimensional organotypic co-culture (3D-OTC) model for HNSCC that maintains the architecture and cell composition of the individual tumor. A dermal equivalent (DE), composed of healthy human-derived fibroblasts and viscose fibers, served as a scaffold for the patient sample. DEs were co-cultivated with 13 vital HNSCC explants (non-human papillomavirus (HPV) driven, *n* = 7; HPV-driven, *n* = 6). Fractionated irradiation was applied to 5 samples (non-HPV-driven, *n* = 2; HPV-driven *n* = 3). To evaluate expression of ki-67, cleaved caspase-3, pan-cytokeratin, p16^INK4a^, CD45, ∝smooth muscle actin and vimentin over time, immunohistochemistry and immunofluorescence staining were performed Patient checkup data were collected for up to 32 months after first diagnosis. *Results*: All non-HPV-driven 3D-OTCs encompassed proliferative cancer cells during cultivation for up to 21 days. Proliferation indices of primaries and 3D-OTCs were comparable and consistent over time. Overall, tumor explants displayed heterogeneous growth patterns (i.e., invasive, expansive, silent). Cancer-associated fibroblasts and leukocytes could be detected for up to 21 days. HPV DNA was detectable in both primary and 3D-OTCs (day 14) of HPV-driven tumors. However, p16^INK4a^ expression levels were varying. Morphological alterations and radioresistant tumor cells were detected in 3D-OTC after fractionated irradiation in HPV-driven and non-driven samples. *Conclusions*: Our 3D-OTC model for HNSCC supports cancer cell survival and proliferation in their original microenvironment. The model enables investigation of invasive cancer growth and might, in the future, serve as a platform to perform sensitivity testing upon treatment to predict therapy response.

## 1. Background

Head and neck squamous cell carcinoma (HNSCC) is characterized by inter- and intra-tumorigenic heterogeneity [1] with a variable response to therapy and a high rate of treatment failure. Therefore, overall survival at 5 years varies between 40% and 52% [2,3]. Besides tobacco and alcohol consumption, infection with high-risk types of human papillomavirus (HPV), in particular HPV 16, is a risk factor for oropharyngeal squamous cell carcinoma (OPSCC) [4]. HPV-driven OPSCC has been characterized as a distinct clinical and molecular entity [5,6]. Reliable molecular biomarkers for early detection of primary and recurrent tumors, as well as prediction of treatment response, in HNSCC are sparse. Therefore, patient stratification into an effective, personalized treatment regimen remains challenging [7]. Hence, there is an unmet need for predictive assays not only for ex ante analysis of the individual patient’s response to standard therapies, but also to investigate the therapeutic efficacy of novel drugs.

One essential requirement for adequate tumor models (Table S1) is the maintenance of the pathophysiological tissue composition since tumorigenesis is governed by complex tumor–matrix interactions [8,9,10,11]. Recently introduced HNSCC organoids from patient-derived cell lines are exclusively based on tumor cells and do not maintain the original microenvironment of the tumor [12]. Patient-derived animal models (PDX models) often show little histological similarity to the initial tumor [13], due to concomitant changes in genetic and epigenetic profiles [14,15]. In particular, the establishment of HPV-driven HNSCC cell lines [16,17] and HPV-induced HNSCC xenografts [18,19] still encounters substantial technical challenges. We previously established an ex vivo tissue culture model for non-HPV-driven HNSCC which preserves the structural architecture and the tumor microenvironment [20,21]. The relatively short cultivation period of this model limited the access to resistant tumor cells after in vitro treatment. Furthermore, it was not feasible to analyze cancer cell invasion and dissemination into surrounding tissue. A promising approach to overcome these limitations are organotypic co-culture models (3D-OTC). The OTC technology was previously developed to elucidate physiologic processes of the skin [22,23,24]. Here, we adopted this model to culture fresh HNSCC tumor explants. This preclinical tumor model allows cultivation of cancer slices for up to 21 days in their original tumor microenvironment and investigation of the clonal expansion of resistant cancer cells upon treatment. The 3D-OTC might serve as an ideal platform to predict tumor behavior, response to treatment, and to stratify individuals who might benefit from established therapies as well as from new concepts of precision medicine.

## 2. Materials and Methods

### 2.1. Patient Collective

Thirteen patients with primary HNSCC were included in this study. Samples were obtained at the Departments of Otorhinolaryngology, Head and Neck Surgery and the Department of Maxillofacial Surgery, Heidelberg University Hospital during surgical resection. Informed consent for tissue culture and data collection was obtained from all patients after review by the ethics board of the Medical Faculty of Heidelberg University (S-206/2011) in accordance with the declaration of Helsinki. Exclusion criteria were distant metastases, as well as previous head and neck irradiation. The cohort comprised different tumor localizations. Five oropharyngeal carcinomas and one hypopharyngeal carcinoma were HPV-driven (p16^INK4a^+ and HPV DNA+) (Figure S1).

### 2.2. Preparation of Dermal Equivalents (DEs)

To create the dermal equivalent (DE), human fibroblasts were cultured on a viscose fiber fabric, that was punched in circles of 11mm in diameter and autoclaved in advance. These constructs grew in 12-well plates with hanging inserts in DMEM medium (4g glucose/l//Ham’s F12: 1:1 (500 mL + 500 mL), 10% FCS, 2% Pen/Strep, hydrocortisone (400 µg/L), ascorbic acid (50 µg/mL), aprotinin (250 E/mL)) [24,25,26] for up to 14 days prior to tumor cultivation, allowing fibroblasts to produce extracellular matrix and hence form solid tissue matrices (DEs).

### 2.3. HNSCC Tissue Preparation and Culture of 3D-OTC

After surgical resection, fresh tissue HNSCC samples were procured immediately and transported to the laboratory within 30 min after resection. Fresh tissue slices of approximately 3 mm thickness were cut with a scalpel and carefully placed on top of the DEs. Samples were cultivated in medium, consisting of 44% DMEM (Bio Whittaker BE 12-604FMB100, #880010, Lonza, Basel, Switzerland), 44% Ham’s F12 medium with L-Glutamine (Biozym BE12-615F7MB006, Art.-Nr.: 880074, Lonza, Basel, Switzerland), 10% fetal bovine serum (Sigma Aldrich, St. Louis, MO, USA), 2% Penicillin/Streptomycin (Biochrom AG, Berlin, Germany #A2213), and 400 µg/L hydrocortisone (H0888-10G, Sigma-Aldrich, St. Louis, MO, USA), supplemented with (1:200) aprotinin (A162.4, Carl Roth GmbH & Co. KG, Karlsruhe, Germany) and (1:2000) ascorbic acid (49752, Sigma-Aldrich, St. Louis, MO, USA). The OTC’s viability was checked daily macroscopically, and media were changed every third day. Part of the unprocessed tumor tissue was immediately formalin-fixed and paraffin-embedded on the day of tumor resection as a control for further experiments. On days 7, 14, and 21, consecutive samples were collected, fixed and embedded in paraffin.

### 2.4. Fractionated Irradiation of 3D-OTC

After having established the 3D-HNSCC-OTC for the first eight specimens of the cohort, the subsequent five samples (non-HPV-driven, *n* = 2; HPV-driven, *n* = 3) were cultured for 5 days without any treatment, and on the sixth day we started daily irradiation (2 Gy) for 5 days using X-RAD 320 (Precision X-Ray, North Branford, CT). OTCs of control cultures were mock-treated, thus kept under the exact same culture conditions without treatment of fractionated irradiation and harvested at the same timepoint (day 14) as treated samples. Treated samples were fixed 96 h after the last fraction of irradiation (day 14) (Figure S6).

### 2.5. Histological, Immunohistochemical and Immunofluorescence Analysis

For histological characterization and depiction of morphological integrity, 5-µm-thick sections were stained with hematoxylin solution modified according to Gill III (Merck KGaA, Darmstadt, Germany) and eosin (Karl Roth GmbH). Immunohistochemical (IHC) analysis was performed as previously described [27]. Primary and secondary antibodies are shown in Table S2. Anti-pan-cytokeratin (PanCK) antibody was applied as an epithelial marker. Proliferation was assessed by an anti-ki-67-antibody, apoptosis by an anti-cleaved caspase-3-antibody. We used the pan-leucocyte marker CD45 for identification of immune cells. p16^INK4a^ expression levels were analyzed in all oropharyngeal HNSCC and samples that were defined as p16^INK4a^-positive during routine diagnostics at the Pathology Department in Heidelberg. Likewise, p16^INk4a^ IHC was performed on the OTC samples.

In order to evaluate growth patterns of tumor cells and integrative tissue components, i.e., fibroblasts, co-immunofluorescence (IF) staining for PanCK and vimentin was performed in all samples. Nuclear staining was performed with DAPI dihydrochloride (D9542 Merck KgA). For further analysis of cancer-associated fibroblasts (CAF) within 3D-HNSCC-OTC, co-IF staining for ∝smooth muscle actin (∝SMA) and vimentin was conducted with DAPI dihydrochloride.

### 2.6. Detection of HPV DNA

Detection of HPV DNA was performed by PCR using BSGP5+/6+ primers [28] that target a consensus region within the viral L1 gene followed by gel electrophoresis allowing for visualization and analysis of the amplified products.

### 2.7. Analysis of Tumor Cell Proportion

We used a semiquantitative method in order to evaluate the tumor cell proportion for primary tumors and 3D-OTC. Cells strongly expressing PanCK were considered as tumor cells and thus, the percentage of cells per visual field, expressing PanCK, was estimated as tumor cell proportion. After analysis of 3–5 visual fields including areas of highest and lowest proportion by two independent observers, mean values and respective standard errors of the mean were calculated and plotted for all tumors and timepoints.

### 2.8. Analysis of Proliferation Indices

Proliferation indices were determined by a semi-quantitative visual method. Tumor cells were considered positive for ki-67 only when clear nuclear staining was detected. The percentage total of positive neoplastic nuclei was estimated after analyzing 3–5 visual fields including areas of highest and lowest positivity. The evaluation was performed by three independent observers. Median values and respective quartiles and ranges of each sample were calculated and plotted. Data point mean values for each tumor and respective timepoint were plotted, standard errors of the mean were calculated and depicted with error bars.

### 2.9. Analysis of the Immune Cell Proprtion

Evaluation of the presence and proportion of vital immune cells was performed by a semiquantitative scoring method. Those cells strongly expressing CD45 and showing nuclei as morphological characteristic of viability were considered as vital immune cells. The proportion of vital immune cells per vision field was divided into negative (0, 0%), low (1, 1–33%), moderate (2, 33–66%) and high (3, 66–100%). Scores were evaluated for 3–5 visual fields including areas of highest and lowest immune cell proportion by two independent observers. Median values and respective standard errors of the mean were calculated and plotted for each tumor and timepoint.

## 3. Results

**Figure 1:**
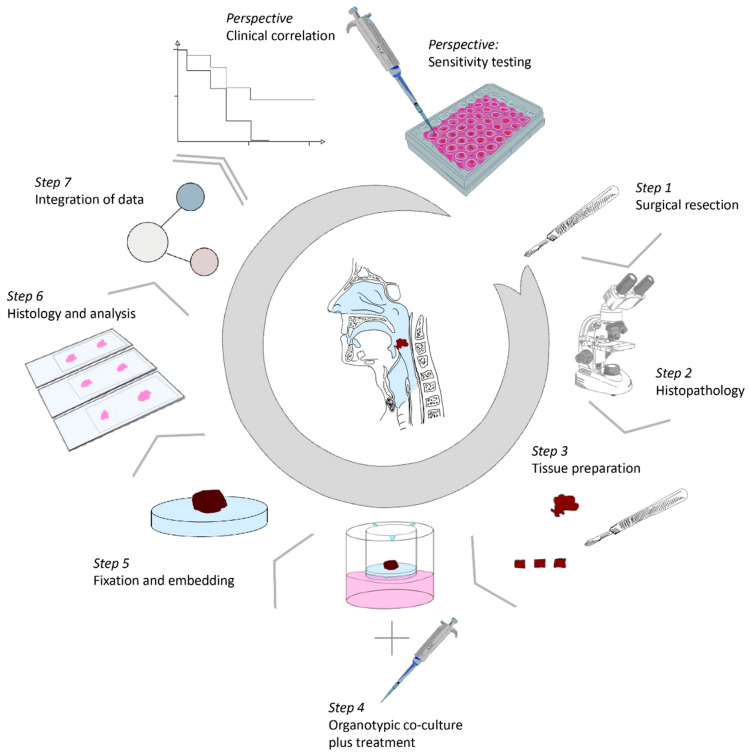
Workflow of the experimental setting. After surgical resection (step 1), vital tumor tissues were examined by a pathologist macroscopically and/or by frozen section technique (step 2) and cut into 2–3-mm-thick slides. Samples were placed on top of the prepared DE (step 3) and kept in culture for up to 21 days (step 4), further harvested, formalin-fixed and paraffin-embedded (step 5). Tissue sections were analyzed by H/E staining and immunohistochemical staining (step 6, 7). When clinical correlation with large cohorts is provided, the 3D-OTC-model offers the perspective of sensitivity drug testing and/or studying the effects of irradiation in order to improve HNSCC patient care. Abbreviations: 3D-OTC, 3D organotypic co-culture; HNSCC, head and neck squamous cell carcinoma.

Thirteen HNSCC samples were cultivated on dermal equivalents for up to 21 days (Figure S1). 3D-HNSCC-OTC were harvested for all 13 HNSCC on day 7 and day 14 respectively. For 7 out of 13 HNSCC samples, one additional 3D-HNSCC-OTC was harvested on day 21. A total of 6 out of 13 HNSCC were cultivated for up to 14 days, due to a pilot phase, in which the experimental setting was designed for 14 days of cultivation (HNSCC1, 2 and 3), detachment of DE (HNSCC10), suspicion of contamination (HNSCC11) or small tumor mass and thus limited material for tissue explants (HNSCC13). After formalin fixation and paraffin embedding, tissue sections were analyzed for proliferation, apoptosis, HPV status (DNA detection and p16^INK4a^ IHC), and post-radiogenic features (Figure 1).

Nine samples were stained for p16^INK4a^ expression during routine diagnostics by the Department of Pathology and 6/9 showed strong and diffuse nuclear and cytoplasmic staining in ≥70% of the tumor. Those six were later confirmed to be HPV-driven by DNA analysis. In the following sections, results for HPV non-driven samples (*n* = 7) are presented separately from HPV-driven HNSCC (*n* = 6).

### 3.1. D-OTC Models from Non-HPV-Driven Tumors

#### 3.1.1. Cancer Cell Viability, Proliferation, and Apoptosis of Non-HPV-Driven HNSCC

**Figure 2:**
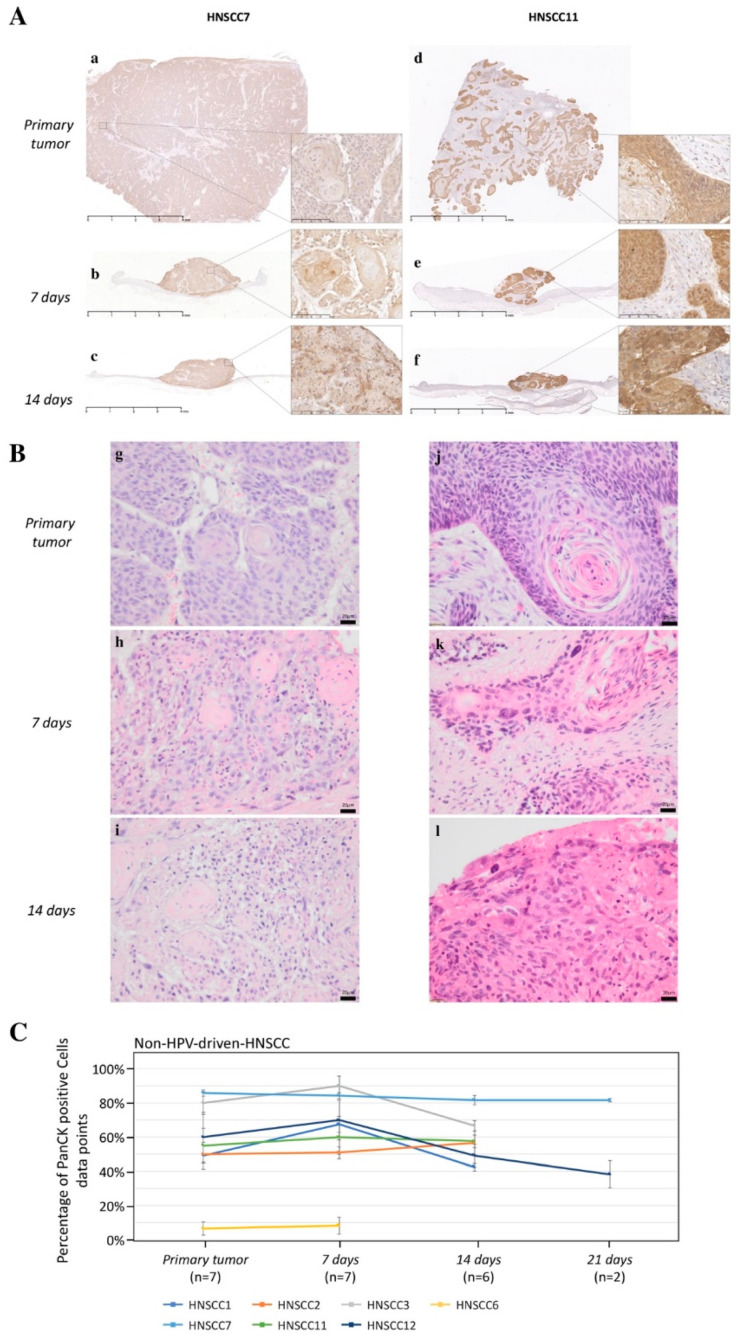
Morphology and PanCK-immunohistochemistry of two non-HPV-driven 3D-OTCs. An anti-PanCK antibody was used to visualize the amount and spatial distribution of PanCK-positive cancer cells (brown signal). For visualization of cellular integrity, details in 16-fold higher magnification are shown for each sample (**A**). Scale Bar in images with lower magnification: 4 mm, and higher magnisfication: 100 µm. (**B**) H/E staining of the according samples. Scale bar: 20 µm. (**C**) Mean values of tumor cell proportion of all primaries and 3D-OTCs on day 7, 14, and 21 of all non-HPV-driven HNSCC. Error bars indicate standard errors of the mean. Abbreviations: PanCK, pan-cytokeratin; HPV, human papillomavirus; 3D-OTC, 3D organotypic co-culture; H/E, hematoxylin and eosin. HNSCC, head and neck squamous cell carcinoma.

**Figure 3:**
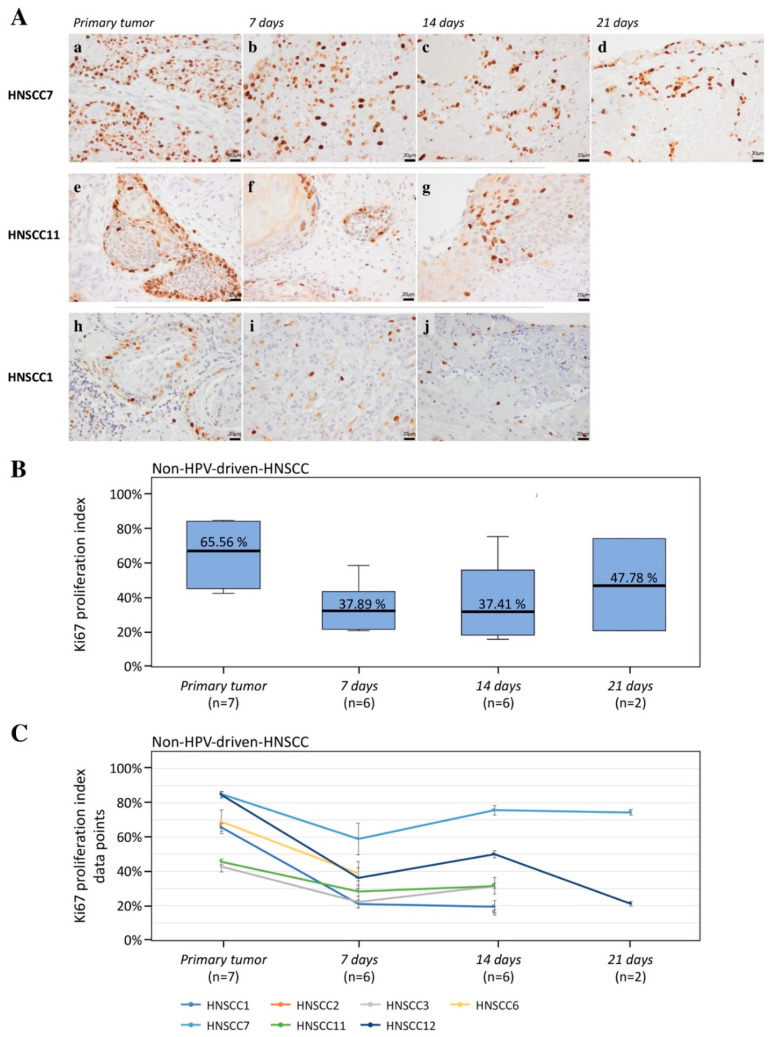
(**A**) Representative pictures of immunohistochemical staining with an anti-ki-67 antibody from three non-HPV-driven 3D-OTCs (b–f,h,i), kept in culture for the indicated periods of time, and corresponding primary tumors (a,d,g) to assess proliferation. HNSCC7 shows strong ki-67 expression in the primary (a). The proliferation index is only minorly reduced up to day 21 (b–d). HNSCC11 presents constant proliferation indices of proliferating tumor cells in primary (e) and 3D-OTCs [19]. Ki-67 expression in HNSCC1′ primary is moderate (h) and presents with a visible decrease of the proliferation index up to day 14 (i,j). Scale Bar: 20 µm. (**B**) Boxplot of ki-67 proliferation indices of primaries and 3D-OTCs on day 7, 14, and 21 of all non-HPV-driven HNSCC. (**C**) Mean values of ki-67 proliferation indices of all primaries and 3D-OTCs on day 7, 14, and 21 of all non-HPV-driven HNSCC. Error bars indicate standard errors of the mean. Abbreviations: HPV, human papillomavirus; 3D-OTC, 3D organotypic co-culture; HNSCC, head and neck squamous cell carcinoma.

The first objective was to detect whether tumor-inherent morphological characteristics were maintained in the 3D-OTC model over time. To assess histological and cellular features, hematoxylin and eosin (H&E) staining was performed and PanCK expression was analyzed by IHC staining on tissue sections from 3D-OTC and primary tumor samples they were derived from. IHC and H&E results confirmed comparable histological and morphological characteristics between primary non-HPV-driven tumors and 3D-OTC over a time period of at least 14 days (Figure 2). The percentage of PanCK positive cells, considered as tumor cell proportion, was stable over time (Figure 2C). Moreover, proliferative activity of cancer cells as determined by ki-67 expression was maintained for up to 21 days in 3D-OTC cultures in all samples (Figure 3). We observed a minor to moderate reduction in the proliferative index in six out of seven OTC samples on day 7 as compared to the primary tumors (mean reduction (MR)—28.67%). Subsequently, the proliferation index remained stable during culture (MR(3D-OTC d14 to d7) + 0.52%, MR(3D-OTC d21 to d14) + 10.34% (Figure 3B,C). In order to analyze the rate of apoptosis over time, we performed IHC for cleaved caspase-3 expression on two non-HPV-driven OTC samples. There was a moderate increase in apoptotic tumor cells in both 3D-OTCs as compared to matched primary tumors (Figure S2).

#### 3.1.2. Motility and Invasiveness of Non-HPV-Driven HNSCC

**Figure 4:**
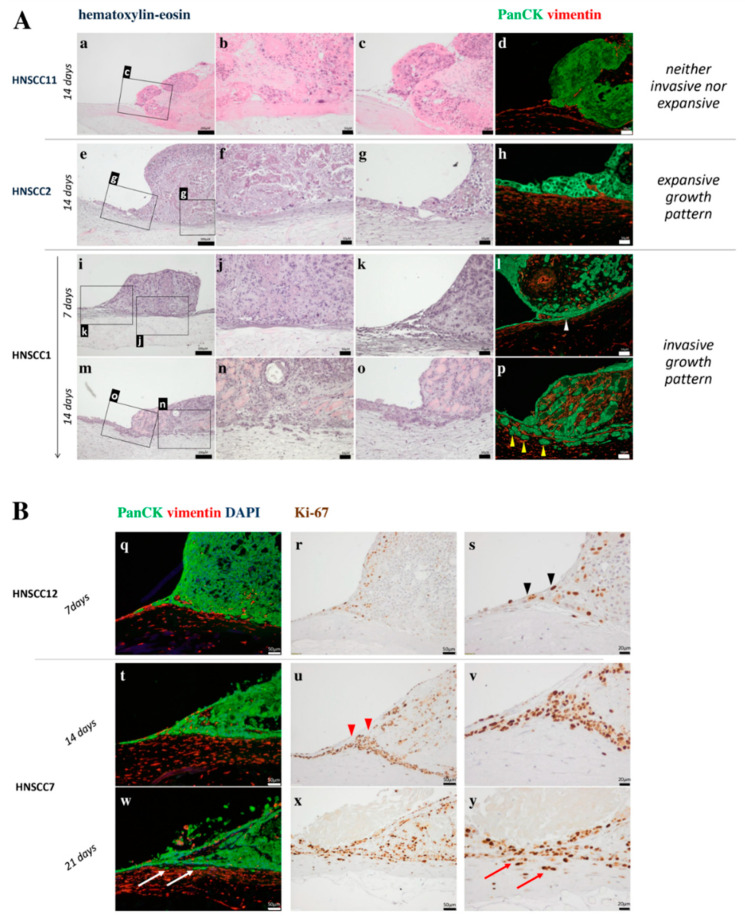
Visualization of distinct tumor growth patterns by H/E staining, IHC, and IF. (**A**) Representative pictures of three different non-HPV-driven 3D-OTCs; culture duration and staining with H/E and IF as indicated in order to visualize growth patterns of the individual sample. HNSCC11 does not show migration or invasion. Tumor cells of HNSCC2 migrate horizontally on top of the dermal equivalent. HNSCC1 presents expansively growing tumor cells on top as well as few detached clusters of tumor cells in the DE on day 7 (i–l; white arrowhead), which impose in larger size and number on day 14 (m–p; yellow arrowheads). Scale Bar: 200 µm (a,e,I,m), 50 µm(b–d,f–h,j–l,n–p). (**B**) IF and IHC with indicated antibodies of two 3D-OTCs at different timepoints. HNSCC12, classified as expansively growing, shows a one-layered migration front (q) and strong proliferation (r,s: black arrowheads). HNSCC7, classified as invasive, presents strong ki-67 expression at the invasive tumor front on day 14 (x,u: red arrowheads) and tumor bulks that invade the DE (w: white flashes), containing high ki-67 levels (x,y). Scale Bar: 50 µm (q,r,t,u,w,x) and 20 µm (s,v,y). Abbreviations: H/E, hematoxylin and eosin; IHC, immunohistochemistry; IF, immunofluorescence; HPV, human papillomavirus; 3D-OTC, 3D organotypic co-culture; DE, dermal equivalent; HNSCC, head and neck squamous cell carcinoma.

Migration patterns at the invasive tumor front (ITF) are known to impact on clinical behavior of HNSCC. Hence, we aimed to address whether the 3D-OTC model allows assessment of tumor cell motility and invasion at different time points. We identified three distinct growth patterns concerning cancer cell motility on day 14. The invasive growth pattern (*n* = 2) was characterized by scattered small irregular clusters of tumor cells invading the DE. Expansively growing samples (*n* = 2) showed horizontal tumor cell spreading along the top of the DE. Silently growing HNSCC (*n* = 3) showed neither invasion nor expansion but exhibited vital tumor tissue over time. ki-67 IHC displayed strong positivity of invasive HNSCC cells. Co-IF staining of PanCK and vimentin showed an ongoing stable expression of cytokeratin until day 21, even within the highly invasive tumor front (Figure 4).

#### 3.1.3. Cancer-Associated Fibroblasts in Non-HPV-Driven HNSCC

**Figure 5:**
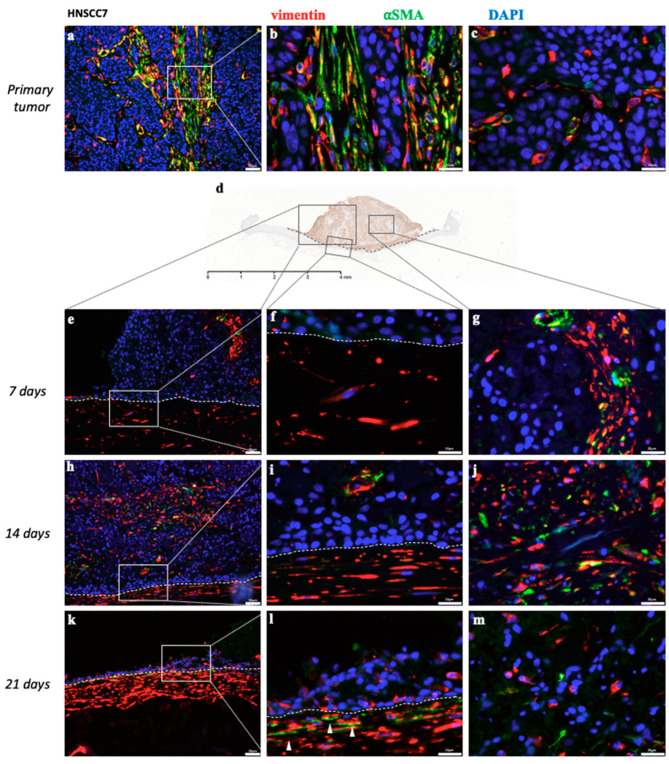
Representative pictures of co-IF staining for vimentin (red), ∝SMA(green) and DAPI of non-HPV-driven HNSCC7 (invasive type) in 3D-OTC (**e***–***m**) and primary tumor (**a**–**c**) as well as an exemplarily overview scan of an IHC staining for PanCK of the according 3D-OTC on day 7 (**d**), that illustrates the localization within the 3D-HNSCC-OTC of the IF images shown below. While (**e**,**h**,**k**) show an overview, (**f**,**i**,**l**) depict in higher magnification the transition of the tumor, planted on top of the DE, and the DE. The border of both compartments is indicated by dotted lines. (**g**,**j**,**m**) show the intratumoral proportion. The primary tumor shows heterogenous ∝SMA expression in different regions (**b**,**c**) and ongoing ∝SMA expression can be detected in intratumoral fibroblasts for up to 21 days in 3D-OTC (**g**,**j**,**m**). The fibroblasts within the DE do not express ∝SMA, but only vimentin on day 7 (**e**,**f**) and day 14 (**h**,**i**). On day 21, those fibroblasts in the DE, that are in direct contact to the ITF, start to express ∝SMA (**l**, see white arrowheads). Scale Bar: 4 mm (**d**); 50 µm (**a**,**e**,**h**,**k**) and 20 µm (**b**,**c**,**f**,**g**,**i**,**j**,**l**,**m**). Abbreviations: IHC, immunohistochemistry; IF, immunofluorescence; ∝SMA, ∝smooth muscle actin; PanCK; pan-cytokeratin; HPV, human papillomavirus; 3D-OTC, 3D organotypic co-culture; DE, dermal equivalent; HNSCC, head and neck squamous cell carcinoma; ITF, invasive tumor front.

As the tumor microenvironment (TME) plays an important role in tumor behavior, progression and metastasis, we aimed to detect CAF in 3D-HNSCC-OTC in order to evaluate whether this model is suited for further TME analysis. Therefore, we performed a co-IF with an anti-∝SMA and an anti-vimentin antibody for the two invasively growing 3D-HNSCC-OTC of non-HPV-driven HNSCC (HNSCC1 and HNSCC7) and according primary tumors. Within the intratumoral proportion of the 3D-HNSCC-OTC planted on top of the DE ∝SMA, positive cells were detected for up to 14 and 21 days (see Figure 5b,c,g,j,m and Figure S9a,b). In general, the DE-inherent human fibroblasts did express only vimentin and no ∝SMA in 3D-HNSCC-OTC. However, those fibroblasts in the DE that were adjacent to regions with infiltrating tumor cells started to express ∝SMA on day 14 (HNSCC1) and day 21 (HNSCC7) (Figure 5l) in the two invasively growing 3D-HNSCC-OTC.

#### 3.1.4. Expression of CD45 as a Pan-Leukocyte Marker in Non-HPV-Driven HNSCC

**Figure 6:**
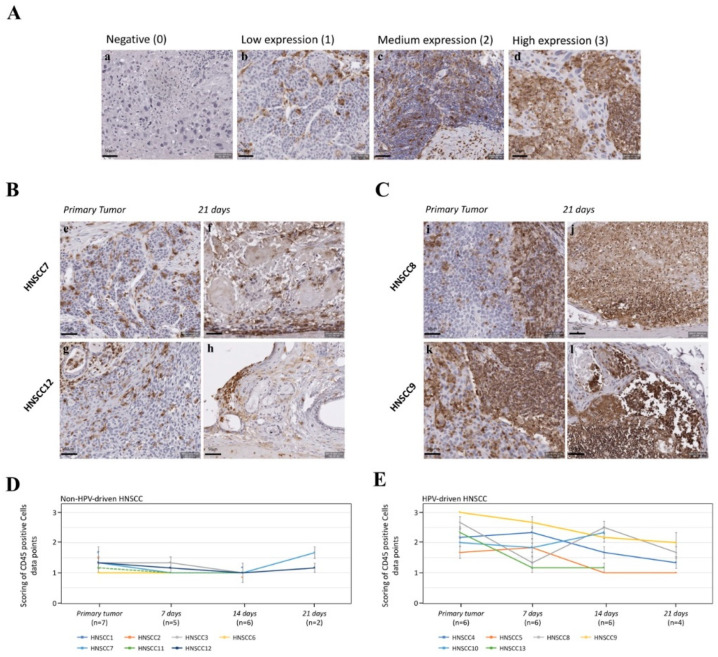
(**A**) Images of IHC with an anti-CD45 antibody. Scoring scale for semiquantitative analysis of immune cell proportion. (a) is negative (0) for CD45, (b) shows low expression (1), (c) moderate expression (2) and d high expression (3) of CD45. (**B**) Representative images of IHC staining of two non-HPV-driven 3D-OTC on day 21 (f,h) and respective primary tumors (e,g) to identify immune cells. HNSCC7 and HNSCC12 show a comparable immune cell proportion. (**C**) Representative images of IHC staining of two HPV-driven 3D-OTC on day 21 (j,l) and respective primary tumors (i,k). HNSCC8 and HNSCC9 show comparably high amounts of immune cells in primaries and 3D-OTC. (**D**) Mean values of scores for CD45-positive, nuclei-positive cells of primaries and 3D-OTCs on day 7, 14, and 21 of all non-HPV-driven HNSCC. Error bars indicate standard errors of the mean. (**E**) Mean values of scores for CD45-positive, nuclei-positive cells of primaries and 3D-OTCs on day 7, 14, and 21 of all HPV-driven HNSCC. Error bars indicate standard errors of the mean. Scale Bar: 50 µm. Abbreviations: HPV, human papillomavirus; 3D-OTC, 3D organotypic co-culture; HNSCC, head and neck squamous cell carcinoma; IHC, immunohistochemistry.

The infiltration of tumor and stroma with immune cells was assessed by CD45 immunohistochemical staining of both non-HPV-driven (*n* = 7) and HPV-driven (*n* = 6) (see Section 3.2.4) HNSCC. We aimed to determine whether infiltration levels were stable during cultivation and consistent in primary tumor and 3D-OTCs. Expression levels were divided into negative (0), low (1), moderate (2) and high (3) expression (Figure 6A). Primaries of non-HPV-driven HNSCC showed low immune cell proportions, that remained stable during culture in 3D-OTC (see Figure 6B,D).

### 3.2. D-OTC Models from HPV-Driven Tumors

#### 3.2.1. Cancer Cell Viability, Proliferation, and Apoptosis of HPV-Driven HNSCC

Six 3D-OTC models were generated from HNSCC, thereof five OPSCC, with strong and diffuse nuclear and cytoplasmatic expression of p16^INK4a^ in more than 70% of the tumor cells as determined during pathological routine examination by IHC. All six p16^INK4a^ overexpressing HNSCC were tested positive for HPV DNA in their primaries as well as in the 3D-OTC at day 14.

**Figure 7:**
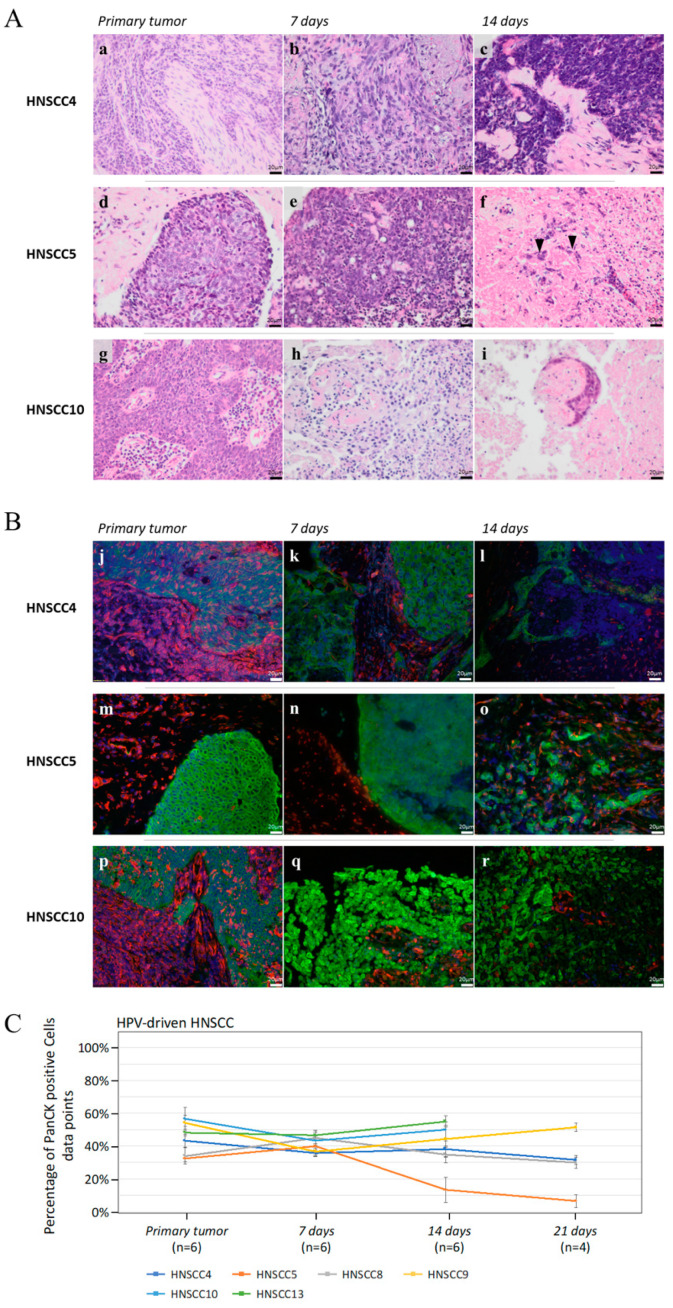
(**A**) H/E staining of three different HPV-driven 3D-HNSCC-OTC (b–f,h,i) and corresponding primaries (a,d,g). HNSCC4 maintains its tumorous morphology. HNSCC5 shows few remaining tumor cells on day 14 (f: black arrowheads) and HNSCC10 presents an altered morphology. Scale Bar: 20 µm. (**B**) Co-immunofluorescence staining with an anti-PanCK-antibody (green), an anti-vimentin-antibody (red), and DAPI (blue) of the samples presented in (**A**), confirming the observations. Scale Bar: 20 µm. (**C**) Mean values of tumor cell proportion of all primaries and 3D-OTCs on day 7, 14, and 21 of all HPV-driven HNSCC. Error bars indicate standard errors of the mean. Abbreviations: H/E, hematoxylin/eosin; HPV, human papillomavirus; 3D-OTC, 3D organotypic co-culture; PanCK, pan-cytokeratin.

**Figure 8:**
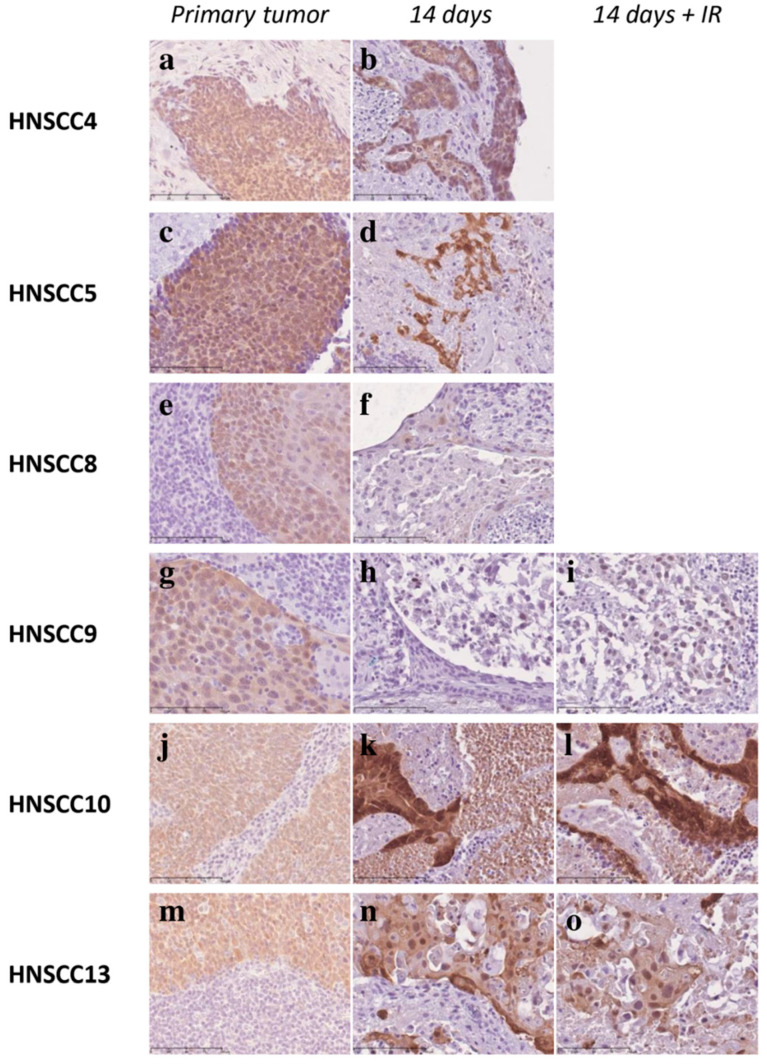
Representative images of an IHC, performed with an anti-p16^INK4a^-antibody, of all six cultured HPV-driven 3D-OTC on day 14 (**b**,**d**,**f**,**h**,**k**,**n**), according primaries (**a**,**c**,**e**,**g**,**j**,**m**) as well as according 3D-OTC on day 14, treated with fractionated irradiation (IR) (**i**,**l**,**o**). While p16^INK4a^ was stably expressed in unirradiated HNSCC4, 5, 10, and 13 up to day 14, there was a strong decrease of expression in HNSCC8 and 9. P16^INK4a^ expression does not differ between irradiated and non-irradiated 3D-OTC on day 14. Scale Bar: 100 µm. Abbreviations: HPV, human papillomavirus; 3D-OTC, 3D organotypic co-culture; IR fractionated irradiation.

Three 3D-OTC displayed comparable tumor morphology at day 14 with matched primary HNSCC (HNSCC4, HNSCC8 and HNSCC13). However, two samples, HNSCC10 and partly HNSCC9, displayed morphological changes and altered tumor cell integrity after 14 days. 3D-OTCs derived from one tumor (HNSCC5) harbored only few tumor cells growing in the 3D-OTC at day 14 and 21, detected by PanCK staining (Figure 7). The percentage of PanCK-positive cells, considered as tumor cell proportion, was evaluated by a semiquantitative method. The evaluation verified the above-mentioned reduction of tumor cells of HNSCC5, whereas the remaining five 3D-OTC of HPV-driven HNSCC were stably expressing PanCK over time (Figure 7C). HPV-driven samples were examined for expression of ki-67. All tumors presented ongoing ki-67 expression up to day 21. Moreover, proliferation indices were examined analogous to non-HPV-driven samples. We observed a moderate mean reduction of proliferation index on day 7 compared to primary (MR= −17.22%), subsequent stable proliferation until day 14 (MR(3D-OTC d14 to d7): −0.00%) and further reduction of proliferation until day 21 (MR(3D-OTC d21 to d14): −17.59%) (Figure S3). Cleaved caspase-3 IHC was performed for three (*n* = 3) HPV-driven HNSCC (HNSCC9, HNSCC10 and HNSCC13) for primary and 3D-OTC, revealing a strong increase of the apoptosis marker over time in two (*n* = 2/3) HNSCC (HNSCC9 and HNSCC10) and stable cleaved caspased-3 expression in one (*n* = 1/3) sample (HNSCC13) (Figure S4). IHC staining confirmed consistent p16^INK4a^ overexpression over time in four out of six HPV-driven 3D-OTC. In two HPV-driven 3D-OTC generated from HNSCC8 and HNSCC9, p16^INK4a^ staining intensity was decreased on day 14 (Figure 8).

#### 3.2.2. Motility and Invasiveness of HPV-Driven HNSCC

We analyzed growth patterns in all six samples of the HPV-driven subgroup as well. Three tumors displayed expansive, and three tumors silent growth patterns, thereof HNSCC5, harboring few remaining tumor cells, as well as HNSCC10, displaying entirely altered tumor cell morphology. HNSCC9, showing only partly altered cell morphology, presented apart from this an extensive expansion-front and was thus ranged among the expansively growing tumors. Interestingly, none of the HPV-driven tumors could be classified as invasive type, in contrast to 3D-OTC from non-HPV-driven samples (Figure S5).

#### 3.2.3. Cancer-Associated Fibroblasts in HPV-Driven HNSCC

In order to evaluate CAF we also performed a co-IF with an anti-∝SMA and an anti-vimentin antibody for two 3D-HNSCC-OTC of HPV-driven HNSCC (HNSCC9 and HNSCC13) and respective primary tumors. We detected an ongoing expression of ∝SMA within the intratumoral fibroblasts in 3D-OTC for up to 21 days (Figure S9c–f).

#### 3.2.4. Expression of CD45 as a Pan-Leukocyte Marker in HPV-Driven HNSCC

Analogical to non-HPV-driven HNSCC, we performed CD45 immunohistochemical staining on HPV-induced primaries and 3D-OTCs and further semiquantitative scoring of the immune cell proportion. Not surprisingly, as most of those tumors originate from the tonsils, primaries of HPV-induced HNSCC were scored higher than primaries of non-HPV-induced HNSCC (Figure 6B,C). All HPV-induced 3D-OTCs presented vital immune cells up to day 21. However, a slight to moderate reduction in the scoring values over time was detected for HPV-driven 3D-OTC (see Figure 6E). Moreover, we observed the presence of CD45-positive regions without morphologically correlating nuclei in 3D-OTC, indicating an in-part death of immune cells.

### 3.3. Fractionated IR of 3D-OTCs 

In order to exemplify the feasibility of a treatment regime, we applied fractionated IR (daily dose of 2 Gy) to five 3D-OTC (*n* = 2 non-HPV-driven, *n* = 3 HPV-driven) models for 5 days (Figure S6). Samples were harvested 4 days after treatment. HPV DNA was detected in all three HPV-driven HNSCC on day 14 after fractionated irradiation. Analysis of p16^INK4a^ expression was performed by IHC staining and displayed in those three samples comparable results to non-irradiated 3D-OTCs of respective primaries on day 14 (Figure 7). Irradiation-induced apoptosis (cleaved caspase-3) and proliferation (ki-67) were analyzed by IHC staining of consecutive tissue sections (Figure S7). As compared to mock-treated controls, irradiated samples revealed heterogeneous proliferation indices in terms of slight increase or decrease of ki-67. Mean overall reduction (MR) of proliferation index, comparing 3D-OTC on day 14 with and without applied fractionated IR, showed stable ki-67 expression after fractionated irradiation (MR = +0.22%), likewise regarding the non-HPV-driven subgroup (MR(3D-OTC d14+fractionated IR to d14 control) = −5.28%) as well as the HPV-driven subgroup (MR(3D-OTC d14+fractionated IR to d14 control) = +3.89%). We investigated post-radiogenic induction of apoptosis by evaluating the expression of cleaved caspase-3 in comparison with mock-treated controls. Interestingly, we were able to observe different responses to irradiation among the five irradiated samples. Three tumors (HPV-driven HNSCC9, non-HPV-driven HNSCC11 and non-HPV-driven HNSCC12) showed similar patterns of cleaved caspase-3 expression in the untreated sample and after irradiation, while in two tumors, (HNSCC10 and HNSCC13, both HPV-driven) apoptotic cells increased after fractionated IR). Non-apoptotic tumor cells were found in clusters. (Figure S8).

### 3.4. Correlation of Patient Relapse and Growth Patterns of 3D-HNSCC OTC 

Routine follow-ups were performed for all patients (*n* = 13) to date for up to 32 months. To our knowledge, at present, all patients are alive. An overview of the clinical data as well as the treatment details is given in Table S3.

The TNM Classification for Malignant Tumors ranged between T1 (*n* = 1), T2 (*n* = 7), T3 (*n* = 2), and T4 (*n* = 3). Seven patients were harboring lymph node metastases, however, no patient was suffering from distant metastases. One patient (HNSCC12) suffered additionally from a lung malignancy, diagnosed a few months after surgery. The patients were treated according to the guidelines for head and neck cancer with modifications if necessary due to individual conditions of the patients.

One patient (*n* = 1/13; HNSCC1) developed local relapse. The corresponding 3D-OTC of HNSCC1 showed an invasive growth pattern. The initial tumor stage was classified as T3N1M0 and the patient underwent tumor resection and neck dissection followed by adjuvant fractionated radiotherapy with a total dosage of 54 Gy. The PFS (progression-free survival) for this patient was 17 months, overall survival (OS) was 32 months. The relapse was treated by revision surgery. The patient has remained disease-free ever since.

## 4. Discussion

Suitable preclinical models for sensitivity testing are a key prerequisite for the establishment and optimization of innovative therapeutic concepts for a tumor entity such as HNSCC with a pronounced inter- and intra-tumorigenic heterogeneity [29,30]. We developed a novel 3D-OTC model for head and neck cancer as an improvement of previously established ex vivo tumor cultures [20,21]. While maintaining physiological tumor–stroma interactions and morphological phenotype, the model allows the characterization of several growth patterns of non-HPV-driven HNSCC samples for up to 21 days. For HPV-driven HNSCC we observed a reduction of tumor cells in one out of six samples (15%) and thus maintenance of tumor tissue in 85% (5/6) of the 3D-OTC and the morphological phenotype was maintained in three out of these five samples (50%) for up to 21 days. For exemplarily applied fractionated irradiation, we demonstrated furthermore the feasibility of treatment testing and response evaluation.

Existing preclinical models for HNSCC such as cell cultures of immortalized tumor cells, conventional or patient-derived xenograft animal models, ex vivo models and recently developed organoids have been introduced during the past decades. All these models have a value for HNSCC research, however none of the existing models can fulfil all the requirements. It is well known that cancer cell lines undergo genetic alterations [31,32] and the success rate of establishing HNSCC cell lines is favored by advanced or metastatic tumor stage and recurrence [33]. This results in a high selective pressure [34,35,36], and thus the intertumoral heterogeneity cannot be depicted entirely. Moreover, success rates in the establishment of HPV-positive HNSCC cell lines are even lower [17,37] and compared to the multitude of established non-HPV induced HNSCC cell lines, only few HPV-driven cell lines exist [16].

Conventional mouse xenograft models [38] as well as patient-derived xenograft (PDX) models [39] show limitations due to the adaption of human tumor cells to the murine microenvironment and the replacement of human stromal components by murine equivalents [40,41]. The costly and time-consuming implementation of other models such as humanized mouse models [42] and orthotopic PDX models [43] also remains sparse for HNSCC [44].

Moreover, the engraftment of HPV-driven HNSCC compared to HPV non-driven HNSCC appears to be complicated as reported in various studies [18,19].

Another challenge of PDX models is the lack of a functional human immune system. The humanized mouse model may overcome this limit [42] but makes its implementation costly and time consuming [39].

One model to study the dissemination of tumor cells in a three-dimensional space is the recently introduced technique of organoid cultivation. However, data on HNSCC and organoids are sparse [12] and Roerink et al. showed a mutational diversification between the primary tumor and its organoids, as well as between different organoids, deriving from the same tumor in colorectal adenocarcinoma [45]. Moreover, the organoid model lacks the ability to depict tumor–stroma interaction and therefore misses displaying the influence of the immune system [46]. Overall, there is an urgent need for a suitable preclinical model for HNSCC that maintains the architecture and the microenvironment of the tumor, is reasonably time- and cost-effective, and thus can be widely implemented, that overcomes the difficulty of culturing non-HPV-driven HNSCC and that can be used to assess long-term treatment effects.

In our study, cell heterogeneity and microenvironment, including vital immune cells, are maintained in the 3D-OTC model. Samples were taken into culture immediately after surgical intervention without any long-lasting time delay and remained viable for up to 21 days, measured by constant expression of PanCK and ki-67. Apoptotic rates also remained stable over time.

Immune cells depicted by CD45 immunohistochemistry were observed in tumor and stroma up to day 21 indicating that 3D-OTC is suitable to monitor tumor–immune interactions over time which is of particular interest for capturing current immunotherapy approaches. The model is also appropriate for addressing the impact of the TME on tumoral features. We were able to detect CAFs in representative primaries and 3D-OTCs of all three growth patterns and from both HPV-driven and non-HPV-driven subgroups for up to 21 days.

Although it was possible to culture the samples of HPV-driven HNSCC up to day 21, two samples showed decreased levels of p16INK4a expression at day 14, when compared to the primary tumor at day 0. This loss of expression might be explained by the clonal expansion and selection of tumor cells in the course of cultivation. Moreover, one of the samples, HNSCC5, showed a decreased amount of cancer cells on day 14. Establishing 3D-OTCs from HPV-driven OPSCC appears to be a challenging task, which is in line with our findings concerning the establishment of HPV-driven HNSCC cell lines and PDX models [16,17,18,19]. However, the success rate of culturing those HPV-driven HNSCC with the 3D-OTC model seems to be higher compared to the establishment of cell lines [17,37] and engraftment rate of PDX [18,19].

Distinct growth patterns were observed in the 3D-OTC model, namely invasive, expansive and silent considering cancer cell motility and invasion, most likely reflecting the heterogeneity of non-HPV induced HNSCC. Invasion patterns at the invasive tumor front have been shown to affect the clinical outcome of HNSCC patients [47,48,49].

Interestingly, the 3D-OTC of the only patient in our cohort suffering from a relapse during follow-up exhibited an invasive growth pattern which might reflect a more aggressive tumor biology. However, due to the small sample size and the rather short follow-up periods, the significance of this finding is limited. Still, if addressed in larger cohorts the 3D-OTC model could pave the way to better understand patterns of cancer cell invasion in a spatial and temporal manner.

The 3D-OTC model enables a more detailed analysis of temporal and spatial response to therapy as exemplified by fractionated IR. There were varying responses characterized by different expression levels of cleaved caspase-3 after five consecutive irradiations. It is known that HNSCC responds rather heterogeneously to standard therapy such as radiation and that HPV-induced HNSCC responds with increased apoptotic rates to radiotherapy compared to non-HPV-driven HNSCC [50]. Our experimental results depict an increase in the expression of cleaved caspase-3 as a marker for apoptosis in only two of five samples after fractionated IR, with both of these tumors being HPV-driven. Interestingly, the non-apoptotic cells of these samples were clustered together. This result might indicate clonally expanding tumor cells. As the number of radiation-treated samples is small, these results may not enable significant deductions but indicate consistency with the current state of knowledge. Thus, we postulate its feasibility of further treatment testing for HNSCC with the 3D-OTC model, including chemotherapy medication as well as immunotherapy. Although we only used samples from primary tumor resections, this model could be of great value for biopsies from patients with unresectable HNSCC in order to assess the patients’ individual risks and stratify them for different treatment regimens.

In summary, 3D-OTCs is a cost-efficient assay to cultivate HNSCC and mimic the response to standard therapy such as irradiation without time delay. Cancer cell dissemination can be detected presumably allowing risk assessment of the individual patient by evaluation of distinct invasion patterns. Moreover, it will eventually enable us to further investigate a resistant subpopulation of tumor cells after treatment. Contamination issues could be minimized by the application of sterile devices during the culturing process. We conclude that the 3D-OTC model is ideally suited to cultivate and examine morphology, invasion, proliferation and apoptosis in non-HPV-driven HNSCC. In HPV-driven HNSCCs, heterogeneous p16^INK4a^ expression indicated a clonal outgrowth although continuous proliferation and apoptosis were detectable, and this observation will be addressed in further HPV-related studies in the future.

## 5. Conclusions

As there is an unmet need for preclinical HNSCC models, especially for non-HPV-driven tumors, which are characterized by poor clinical outcome, we consider the 3D-OTC model to be ideally suited to mimic the tumor in its entirety and to test individual long-term tumor responses to standard treatment or targeted therapies. The model may serve as a promising tool for risk assessment and for patient stratification.

## Supplementary Materials

The following are available online at https://www.mdpi.com/2072-6694/12/8/2330/s1, Figure S1. Specimen origin and experimental setting. (A) Depiction of specimen origin, HPV status, treatment with fractionated IR and growth pattern of 3D-OTC. (B) Details of HPV status and time in culture of 3D-OTC. Abbreviations: IR, irradiation; HPV, human papillomavirus; 3D-OTC, 3D organotypic co-culture, Figure S2. Visualization of apoptosis at different time points. Representative images of immunohistochemistry with an anti-cleaved caspase-3 antibody of two different non-HPV-driven 3D-OTC cultures (*b*–*c* and *e*–*f*) and matching primary tumors (*a* and *d*) for visualization of apoptosis. HNSCC12 shows low levels of apoptotic cells in the primary with a slight increase during cultivation (see black arrowheads). HNSCC11, constantly low expression of cleaved caspase-3 presents in the primary tumor and in 3D-OTC cultures. Abbreviations: HPV, human papillomavirus; 3D-OTC, 3D organotypic co-culture, Figure S3. (A) IHC with an anti-ki-67-antibody of three different HPV-driven 3D-HNSCC-OTC (*b*–*c*, *e*–*f*, *h*–*i*) for the indicated time points and according primaries (*a*, *d*, *g*). (B) Boxplot of ki-67 proliferation indices of primaries and 3D- OTCs on day 7, 14, and 21 of all HPV-driven HNSCC. (C) Mean values of ki-67 proliferation indices of all primaries and 3D-OTCs on day 7, 14, and 21 of all HPV-driven HNSCC. Error bars indicate standard errors of the mean. Abbreviations: IHC, immunohistochemistry; HNSCC, Head and neck squamous cell carcinoma; 3D-OTC, 3D organotypic co-culture, Figure S4. IHC with an anti-cc-3 antibody of three HPV-driven 3D-HNSCC-OTC on day 14 (*b*, *d*, *f*) and according primaries (*a*, *c*, *e*). HNSCC9 and HNSCC10 present an increase of cc-3 expression in 3D-HNSCC-OTC, compared to their primary. HNSCC13 maintains stable expression of cc-3 during culture. Abbreviations: IHC, immunohistochemistry; HPV, human papillomavirus; 3D-OTC, 3D organotypic co-culture; cc-3, cleaved caspase-3, Figure S5. Co-immunofluorescence staining with an anti-PanCK-antibody (green), an anti-vimentin-antibody (red) and DAPI (blue) (*a*–*b*; *d*–*e*) and H/E staining (*c*, *f*) of two different HPV-driven 3D-OTC on day 14. HNSCC8 presents an expansive growth pattern, white arrowheads (a) indicate the MF, HNSCC13 shows a silent growth pattern. Abbreviations: PanCK, pan-cytokeratin; H/E, Hematoxylin/eosin; HPV, human papillomavirus; 3D-OTC, 3D organotypic co-culture; MF, migration front, Figure S6. Diagram showing treatment with fractionated IR. After 5 days in culture 5 HNSCC-OTC were irradiated with 2 Gy on 5 consecutive days followed by 3 holidays before being harvested on day 14. Controls were mock-treated. Abbreviations: IR, irradiation; HNSCC, head and neck squamous cell carcinoma; 3D-OTC, 3D organotypic co-culture, Figure S7. Selection of representative images of H/E, IF and IHC with indicated antibodies of one 3D-OTC, which was cultured for 14 days and treated with mock irradiation (*a*–*g*) or a fractionated irradiation scheme (*h*–*n*), in order to depict radiogenic impact. PanCK-vimentin-Co-IF reveals a reduction of layering in the migration front after fractionated irradiation (see yellow flashes) in comparison to the untreated sample. ki-67-IHC detects stable postradiogenic proliferation and IHC while cleaved caspase-3 shows heterogenous expression in the mock-treated and fractionated irradiated OTC. Apoptotic tumor cells (see red arrowhead) and those, not undergoing apoptosis (see black arrowhead). Abbreviations: H/E, hematoxylin/eosin; IF, immunofluorescence; IHC, immunohistochemistry; 3D-OTC, 3D organotypic co-culture; PanCK, pan-cytokeratin; IR, irradiation, Figure S8. Impact of fractionated irradiation on apoptosis. Representative pictures of IHC with an anti-cc-3 antibody on 3D-OTC (all on day 14) of two different tumors; mock-(*a* and *c*) and matching fractionated-irradiated samples (*b* and *d*), respectively. HNSCC11 shows similar intensity and distribution of the cc-3 signal in the untreated OTC as well as after fractionated IR. Increasing cc-3 expression of fractional irradiated HNSCC13 compared to the mock-treated correlate. Abbreviations: IHC, immunohistochemistry; cc-3, cleaved caspase-3; 3D-OTC, 3D organotypic co-culture; IR, irradiation, Figure S9. Representative pictures of co-IF staining for vimentin (red), ∝SMA (green) and DAPI (blue) of non-HPV-driven HNSCC1 (invasive type) and two HPV-driven HNSCC (HNSCC9; expansive type and HNSCC13; silent type in 3D-OTC on day 14 (*c*, *d*, *e*) and according primary tumor (*a*, *c*, *e*). Respective samples show a similar amount of ∝SMA positive cells in all primaries and according 3D-OTC on day 14. Abbreviations: IF, immunofluorescence; ∝SMA, ∝smooth muscle actin; HPV, human papillomavirus; HNSCC, Head and neck squamous cell carcinoma. Table S1. Currently available preclinical models for HNSCC. Abbreviations: HNSCC, Head and neck squamous cell carcinoma; HPV, human papillomavirus, Table S2. Primary and secondary antibodies for IHC and IF, Table S3. Clinical data and follow ups of patient collective.

## Additional Information

Ethics approval and consent to participate: Informed consent was obtained from all patients after review of the local ethics board (S-206/2011).

## References

[1] Mroz E.A., Rocco J.W. (2016). Intra-tumor heterogeneity in head and neck cancer and its clinical implications. World J. Otorhinolaryngol. Head Neck Surg..

[2] Gatta G., Botta L., Sanchez M.J., Anderson L.A., Pierannunzio D., Licitra L., Group E.W. (2015). Prognoses and improvement for head and neck cancers diagnosed in Europe in early 2000s: The EUROCARE-5 population-based study. Eur. J. Cancer.

[3] Giraldi L., Leoncini E., Pastorino R., Wunsch-Filho V., de Carvalho M., Lopez R., Cadoni G., Arzani D., Petrelli L., Matsuo K. (2017). Alcohol and cigarette consumption predict mortality in patients with head and neck cancer: A pooled analysis within the International Head and Neck Cancer Epidemiology (INHANCE) Consortium. Ann. Oncol..

[4] Gillison M.L., Chaturvedi A.K., Anderson W.F., Fakhry C. (2015). Epidemiology of Human Papillomavirus-Positive Head and Neck Squamous Cell Carcinoma. J. Clin. Oncol..

[5] Miller D.L., Davis J.W., Taylor K.H., Johnson J., Shi Z., Williams R., Atasoy U., Lewis J.S., Stack M.S. (2015). Identification of a human papillomavirus-associated oncogenic miRNA panel in human oropharyngeal squamous cell carcinoma validated by bioinformatics analysis of the Cancer Genome Atlas. Am. J. Pathol..

[6] Spence T., Bruce J., Yip K.W., Liu F.F. (2016). HPV Associated Head and Neck Cancer. Cancers.

[7] Dahiya K., Dhankhar R. (2016). Updated overview of current biomarkers in head and neck carcinoma. World J. Methodol..

[8] Affolter A., Schmidtmann I., Mann W.J., Brieger J. (2013). Cancer-associated fibroblasts do not respond to combined irradiation and kinase inhibitor treatment. Oncol. Rep..

[9] Fischer H., Taylor N., Allerstorfer S., Grusch M., Sonvilla G., Holzmann K., Setinek U., Elbling L., Cantonati H., Grasl-Kraupp B. (2008). Fibroblast growth factor receptor-mediated signals contribute to the malignant phenotype of non-small cell lung cancer cells: Therapeutic implications and synergism with epidermal growth factor receptor inhibition. Mol. Cancer Ther..

[10] Guleng B., Tateishi K., Ohta M., Kanai F., Jazag A., Ijichi H., Tanaka Y., Washida M., Morikane K., Fukushima Y. (2005). Blockade of the stromal cell-derived factor-1/CXCR4 axis attenuates in vivo tumor growth by inhibiting angiogenesis in a vascular endothelial growth factor-independent manner. Cancer Res..

[11] Kim S.Y., Lee C.H., Midura B.V., Yeung C., Mendoza A., Hong S.H., Ren L., Wong D., Korz W., Merzouk A. (2008). Inhibition of the CXCR4/CXCL12 chemokine pathway reduces the development of murine pulmonary metastases. Clin. Exp. Metastasis.

[12] Tanaka N., Osman A.A., Takahashi Y., Lindemann A., Patel A.A., Zhao M., Takahashi H., Myers J.N. (2018). Head and neck cancer organoids established by modification of the CTOS method can be used to predict in vivo drug sensitivity. Oral Oncol..

[13] Russo M.V., Faversani A., Gatti S., Ricca D., Del Gobbo A., Ferrero S., Palleschi A., Vaira V., Bosari S. (2015). A new mouse avatar model of non-small cell lung cancer. Front. Oncol..

[14] Daniel V.C., Marchionni L., Hierman J.S., Rhodes J.T., Devereux W.L., Rudin C.M., Yung R., Parmigiani G., Dorsch M., Peacock C.D. (2009). A primary xenograft model of small-cell lung cancer reveals irreversible changes in gene expression imposed by culture in vitro. Cancer Res..

[15] Morgan K.M., Riedlinger G.M., Rosenfeld J., Ganesan S., Pine S.R. (2017). Patient-Derived Xenograft Models of Non-Small Cell Lung Cancer and Their Potential Utility in Personalized Medicine. Front. Oncol..

[16] Tang A.L., Hauff S.J., Owen J.H., Graham M.P., Czerwinski M.J., Park J.J., Walline H., Papagerakis S., Stoerker J., McHugh J.B. (2012). UM-SCC-104: A new human papillomavirus-16-positive cancer stem cell-containing head and neck squamous cell carcinoma cell line. Head Neck.

[17] Forslund O., Sugiyama N., Wu C., Ravi N., Jin Y., Swoboda S., Andersson F., Bzhalava D., Hultin E., Paulsson K. (2019). A novel human in vitro papillomavirus type 16 positive tonsil cancer cell line with high sensitivity to radiation and cisplatin. BMC Cancer.

[18] Facompre N.D., Sahu V., Montone K.T., Harmeyer K.M., Nakagawa H., Rustgi A.K., Weinstein G.S., Gimotty P.A., Basu D. (2017). Barriers to generating PDX models of HPV-related head and neck cancer. Laryngoscope.

[19] Klinghammer K., Raguse J.D., Plath T., Albers A.E., Joehrens K., Zakarneh A., Brzezicha B., Wulf-Goldenberg A., Keilholz U., Hoffmann J. (2015). A comprehensively characterized large panel of head and neck cancer patient-derived xenografts identifies the mTOR inhibitor everolimus as potential new treatment option. Int. J. Cancer.

[20] Affolter A., Muller M.F., Sommer K., Stenzinger A., Zaoui K., Lorenz K., Wolf T., Sharma S., Wolf J., Perner S. (2016). Targeting irradiation-induced mitogen-activated protein kinase activation in vitro and in an ex vivo model for human head and neck cancer. Head Neck.

[21] Freudlsperger C., Horn D., Weissfuss S., Weichert W., Weber K.J., Saure D., Sharma S., Dyckhoff G., Grabe N., Plinkert P. (2015). Phosphorylation of AKT(Ser473) serves as an independent prognostic marker for radiosensitivity in advanced head and neck squamous cell carcinoma. Int. J. Cancer.

[22] Burnworth B., Popp S., Stark H.J., Steinkraus V., Brocker E.B., Hartschuh W., Birek C., Boukamp P. (2006). Gain of 11q/cyclin D1 overexpression is an essential early step in skin cancer development and causes abnormal tissue organization and differentiation. Oncogene.

[23] Nischt R., Schmidt C., Mirancea N., Baranowsky A., Mokkapati S., Smyth N., Woenne E.C., Stark H.J., Boukamp P., Breitkreutz D. (2007). Lack of nidogen-1 and -2 prevents basement membrane assembly in skin-organotypic coculture. J. Investig. Dermatol..

[24] Stark H.J., Willhauck M.J., Mirancea N., Boehnke K., Nord I., Breitkreutz D., Pavesio A., Boukamp P., Fusenig N.E. (2004). Authentic fibroblast matrix in dermal equivalents normalises epidermal histogenesis and dermoepidermal junction in organotypic co-culture. Eur. J. Cell Biol..

[25] Boehnke K., Mirancea N., Pavesio A., Fusenig N.E., Boukamp P., Stark H.J. (2007). Effects of fibroblasts and microenvironment on epidermal regeneration and tissue function in long-term skin equivalents. Eur. J. Cell Biol..

[26] Pinto S., Stark H.J., Martin I., Boukamp P., Kyewski B. (2015). 3D Organotypic Co-culture Model Supporting Medullary Thymic Epithelial Cell Proliferation, Differentiation and Promiscuous Gene Expression. J. Vis. Exp..

[27] Thierauf J., Weissinger S.E., Veit J.A., Affolter A., Laureano N.K., Beutner D., Heiduschka G., Kadletz L., Meyer M., Quaas A. (2018). Low SOX2 expression marks a distinct subset of adenoid cystic carcinoma of the head and neck and is associated with an advanced tumor stage. PLoS ONE.

[28] Schmitt M., Dondog B., Waterboer T., Pawlita M. (2008). Homogeneous amplification of genital human alpha papillomaviruses by PCR using novel broad-spectrum GP5+ and GP6+ primers. J. Clin. Microbiol..

[29] Perri F., Pacelli R., Della Vittoria Scarpati G., Cella L., Giuliano M., Caponigro F., Pepe S. (2015). Radioresistance in head and neck squamous cell carcinoma: Biological bases and therapeutic implications. Head Neck.

[30] Psyrri A., Seiwert T.Y., Jimeno A. (2013). Molecular pathways in head and neck cancer: EGFR, PI3K, and more. Am. Soc. Clin. Oncol. Educ. Book.

[31] Smiraglia D.J., Rush L.J., Fruhwald M.C., Dai Z., Held W.A., Costello J.F., Lang J.C., Eng C., Li B., Wright F.A. (2001). Excessive CpG island hypermethylation in cancer cell lines versus primary human malignancies. Hum. Mol. Genet..

[32] Suter C.M., Norrie M., Ku S.L., Cheong K.F., Tomlinson I., Ward R.L. (2003). CpG island methylation is a common finding in colorectal cancer cell lines. Br. J. Cancer.

[33] Carey T.E., Hay R.J., Park J.-G., Gazdar A. (1994). Atlas of Human Tumor Cell Lines.

[34] Easty D.M., Easty G.C., Carter R.L., Monaghan P., Butler L.J. (1981). Ten human carcinoma cell lines derived from squamous carcinomas of the head and neck. Br. J. Cancer.

[35] Kim S.Y., Chu K.C., Lee H.R., Lee K.S., Carey T.E. (1997). Establishment and characterization of nine new head and neck cancer cell lines. Acta Otolaryngol..

[36] Sacks P.G., Parnes S.M., Gallick G.E., Mansouri Z., Lichtner R., Satya-Prakash K.L., Pathak S., Parsons D.F. (1988). Establishment and characterization of two new squamous cell carcinoma cell lines derived from tumors of the head and neck. Cancer Res..

[37] White J.S., Weissfeld J.L., Ragin C.C., Rossie K.M., Martin C.L., Shuster M., Ishwad C.S., Law J.C., Myers E.N., Johnson J.T. (2007). The influence of clinical and demographic risk factors on the establishment of head and neck squamous cell carcinoma cell lines. Oral Oncol..

[38] Dohmen A.J., Swartz J.E., Van Den Brekel M.W., Willems S.M., Spijker R., Neefjes J., Zuur C.L. (2015). Feasibility of Primary Tumor Culture Models and Preclinical Prediction Assays for Head and Neck Cancer: A Narrative Review. Cancers.

[39] Choi S.Y., Lin D., Gout P.W., Collins C.C., Xu Y., Wang Y. (2014). Lessons from patient-derived xenografts for better in vitro modeling of human cancer. Adv. Drug Deliv. Rev..

[40] Peng S., Creighton C.J., Zhang Y., Sen B., Mazumdar T., Myers J.N., Lai S.Y., Woolfson A., Lorenzi M.V., Bell D. (2013). Tumor grafts derived from patients with head and neck squamous carcinoma authentically maintain the molecular and histologic characteristics of human cancers. J. Transl. Med..

[41] Cassidy J.W., Caldas C., Bruna A. (2015). Maintaining Tumor Heterogeneity in Patient-Derived Tumor Xenografts. Cancer Res.

[42] Morton J.J., Bird G., Keysar S.B., Astling D.P., Lyons T.R., Anderson R.T., Glogowska M.J., Estes P., Eagles J.R., Le P.N. (2016). XactMice: Humanizing mouse bone marrow enables microenvironment reconstitution in a patient-derived xenograft model of head and neck cancer. Oncogene.

[43] Hoffman R.M. (2015). Patient-derived orthotopic xenografts: Better mimic of metastasis than subcutaneous xenografts. Nat. Rev. Cancer.

[44] Nitschinsk K., Idris A., McMillan N. (2018). Patient derived xenografts as models for head and neck cancer. Cancer Lett..

[45] Roerink S.F., Sasaki N., Lee-Six H., Young M.D., Alexandrov L.B., Behjati S., Mitchell T.J., Grossmann S., Lightfoot H., Egan D.A. (2018). Intra-tumour diversification in colorectal cancer at the single-cell level. Nature.

[46] Mery B., Rancoule C., Guy J.B., Espenel S., Wozny A.S., Battiston-Montagne P., Ardail D., Beuve M., Alphonse G., Rodriguez-Lafrasse C. (2017). Preclinical models in HNSCC: A comprehensive review. Oral Oncol..

[47] Bryne M., Koppang H.S., Lilleng R., Kjaerheim A. (1992). Malignancy grading of the deep invasive margins of oral squamous cell carcinomas has high prognostic value. J. Pathol..

[48] Odell E.W., Jani P., Sherriff M., Ahluwalia S.M., Hibbert J., Levison D.A., Morgan P.R. (1994). The prognostic value of individual histologic grading parameters in small lingual squamous cell carcinomas. The importance of the pattern of invasion. Cancer.

[49] Zhu Y., Liu H., Xie N., Liu X., Huang H., Wang C., Hou J. (2019). Impact of tumor budding in head and neck squamous cell carcinoma: A meta-analysis. Head Neck.

[50] Ziemann F., Arenz A., Preising S., Wittekindt C., Klussmann J.P., Engenhart-Cabillic R., Wittig A. (2015). Increased sensitivity of HPV-positive head and neck cancer cell lines to x-irradiation +/− Cisplatin due to decreased expression of E6 and E7 oncoproteins and enhanced apoptosis. Am. J. Cancer Res..

