# Supplementary material for: Organotypic Co-Cultures as a Novel 3D Model for Head and Neck Squamous Cell Carcinoma
Source: Cancers (Basel). 2020 Aug 18;12(8):2330. doi: 10.3390/cancers12082330 (PMC7463661; doi:10.3390/cancers12082330)
Supplement: Supplementary file 1 [file cancers-12-02330-s001.pdf]

Article

Organotypic Co-Cultures as a Novel 3D Model for Head and Neck Squamous Cell Carcinoma

Luca Engelmann [1,†], Julia Thierauf [1,2,†], Natalia Koerich Laureano [1,3,4], Hans-Juergen Stark [5,6], Elena-Sophie Prigge [5,6], Dominik Horn [7], Kolja Freier [7], Niels Grabe [8], Chao Rong [1,9], Philippe Federspil [1], Karim Zaoui [1], Peter K. Plinkert [1], Nicole Rotter [10], Magnus von Knebel Doeberitz [5,6], Jochen Hess [1,4] and Annette Affolter [1,10,*]

[1] Department of Otorhinolaryngology, Head and Neck Surgery, Experimental Head and Neck Oncology, Heidelberg University Hospital, Im Neuenheimer Feld 400, 69120 Heidelberg, Germany; engelmann.luca@yahoo.de (L.E.); jthierauf@mgh.harvard.edu (J.T.); nataliakoerich@hotmail.com (N.K.L.); rongchaochina@163.com (C.R.); federspil@med.uni-heidelberg.de (P.F.); karim.zaoui@med.uni-heidelberg.de (K.Z.); peter.plinkert@med.uni-heidelberg.de (P.K.P.); j.hess@dkfz-heidelberg.de (J.H.)
[2] Department of Pathology, Massachusetts General Hospital, 55 Fruit Street, Boston, MA02114. USA
[3] Oral Pathology, Federal University of Rio Grande do Sul, Av. Paulo Gama, 110 Porto Alegre, 90040-060, Brazil
[4] Molecular Mechanisms of Head and Neck Tumors, German Cancer Research Center, Im Neuenheimer Feld 280, 69120 Heidelberg, Germany
[5] Department of Applied Tumor Biology, Institute of Pathology, University of Heidelberg, Im Neuenheimer Feld 224, 69120 Heidelberg, Germany; hj.stark@dkfz-heidelberg.de (H.-J.S.); elena.prigge@med.uni-heidelberg.de (E.-S.P.); magnus.knebel-doeberitz@med.uni-heidelberg.de (M.v.K.D.)
[6] Clinical Cooperation Unit Applied Tumor Biology, German Cancer Research Centre, Im Neuenheimer Feld 280, 69120 Heidelberg, Germany
[7] Department of Oral and Maxillofacial Surgery, Saarland University Medical Center, Kirrberger Strasse, 66424 Homburg, Germany; dominik.horn@uks.eu
[8] National Center for Tumor Diseases, Hamamatsu TIGA Center, Bioquant, Heidelberg University, Im Neuenheimer Feld 267, 69120 Heidelberg, Germany; niels.grabe@bioquant.uni-heidelberg.de
[9] Department of Pathology, School of Biology & Basic Medical Sciences, Soochow University, 199 Ren-Ai Road, Suzhou Industrial Park, Suzhou, 215123, China
[10] Department of Otorhinolaryngology, Head and Neck Surgery, University Hospital Mannheim, Medical Faculty Mannheim of Heidelberg University, Theodor-Kutzer-Ufer 1-3, 68167 Mannheim, Germany; Nicole.Rotter@umm.de
* Correspondence: annette.affolter@umm.de
† These authors contributed equally to this work

Supplementary Materials

Table S1. Currently available preclinical models for HNSCC.

Tumour / Derivation	Non-HPV induced HNSCC	HPV-induced HNSCC
Cell culture	**More than 370 HNSCC cell lines**, reviewed by Lin et al, 2007[48]	Steenbergen et al, 1995<br>Ferris et al, 2005<br>White et al, 2007<br>Hoffmann et al, 2008<br>Sartor et al, 2011<br>Tang et al, 2012<br>Forslund et al, 2019
Animal model	Peng et al, 2013[53]<br>Kimple et al, 2013[54]<br>Klinghammer et al, 2014[19]<br>Li et al, 2016[55]<br>Morton et al, 2016[56]<br>Facompre et al, 2017[18]<br>Karamboulas et al, 2018[57]<br>Ruicci et al, 2019[58]	Kimple et al, 2013<br>Klinghammer et al, 2014<br>Facompre et al, 2017

Human, "personalized patient-derived"	Gerlach et al, 2014[40]<br>Tanaka et al, 2019[12]<br>Driehuis et al, 2019[59]<br>Karakasheva et al, 2020[60]<br>Engelmann et al, 2020	Tanaka et al, 2019<br>Engelmann et al, 2020

Abbreviations: HNSCC, Head and neck squamous cell carcinoma; HPV, human papillomavirus.

Table S2. Primary and secondary antibodies for IHC and IF. Abbreviations: IF, immunofluorescence; IHC, immunohistochemistry; cc-3, cleaved caspase-3; PanCK, pan-cytokeratin.

Method	Primary antibody	Ref#	Manufacturer	Dilution	Secondary antibody	Ref#	Manufacturer	Dilution
IHC	Anti-CD45	M0703	Dako, Denmark	1:100	HRP anti-	MP7402	Vector Laboratories, USA	ready to use
IHC	Anti-cleaved caspase-3	9661	Cellsignaling, USA	1:300	Biotinylated anti-rabbit	BA-1000	Vector Laboratories, USA	1:200
IHC	Anti-ki-67	M7420	Dako, Denmark	1:50	HRP anti-mouse	MP7402	Vector Laboratories, USA	ready to use
IHC	Anti-PanCK	GP14	Progen, Germany	1:200	Biotinylated anti-guinea-pig	BA-7000	Vector Laboratories, USA	1:200
IF	Anti-PanCK	GP14	Progen, Germany	1:50	Alexa Fluor488-anti-guinea-pig	ab150185	Abcam, UK	1:200
IHC	Anti-p16 ^{INK4a}	705-4713	Roche, Germany	ready to use	HRP anti-mouse	MP7402	Vector Laboratories, USA	ready to use
IF	Anti-vimentin	61013	Progen, Germany	1:100	Cy3–anti-mouse	715165151	Jackson Immuno Research, UK	1:200

Table S3. Clinical data of patient collective. Abbreviations: Y, years; HPV, human papillomavirus; Gy, Gray; pos, positive; neg, negative; n/a, not applicable; TNM, TNM Classification of Malignant Tumors.

sample #	age by time of surgery	localisation	HPV-Status	TNM	adjuvant Radiotherapy (dose)	adjuvant Chemo-therapy	last check up (after surgery)	timing of first indication of relapse (after surgery)	details	invasion pattern (3D-OTC)
HNSCC1	72 Y	Oral Cavity	n/a	T3N1M0	yes (54 Gy)	no	32 months	17 months	local recurrence	invasive
HNSCC2	60 Y	Oral Cavity	n/a	T4aN0M0	yes (n/a)	no	20 months	n/a	no evidence of disease	expansive
HNSCC3	84 Y	Oral Cavity	n/a	T2N0M0	no	no	18 months	n/a	no evidence of disease	silent
HNSCC4	58 Y	Tonsil	pos	T2N1M0	yes (54 Gy)	yes	29 months	n/a	no evidence of disease	expansive
HNSCC5	65 Y	Tonsil	pos	T2N1M0	no	no	21 months	n/a	no evidence of disease	silent
HNSCC6	67 Y	Tonsil	neg	T2N2aM0	no	no	7 months	n/a	no evidence of disease	silent
HNSCC7	79 Y	Nasal Cavity	n/a	T4aN0M0	no	no	13 months	n/a	no evidence of disease	invasive
HNSCC8	47 Y	Tonsil	pos	T2N0M0	no	no	14 months	n/a	no evidence of disease	expansive
HNSCC9	64 Y	Tonsil	pos	T1N1M0	yes (54 Gy)	no	22 months	n/a	no evidence of disease	expansive
HNSCC10	56 Y	Hypopharynx	pos	T2N0M0	no	no	14 months	n/a	no evidence of disease	silent
HNSCC11	51 Y	Larynx	neg	T4aN1M0	yes (66 Gy)	yes	21 months	n/a	no evidence of disease	silent
HNSCC12	63 Y	Tonsil	neg	T3N0MX	n/a	no	20 months	n/a	no evidence of disease	expansive
HNSCC13	74 Y	Tonsil	pos	T2N1M0	yes (57,6 Gy)	yes	20 months	n/a	no evidence of disease	silent

cancers MDPI

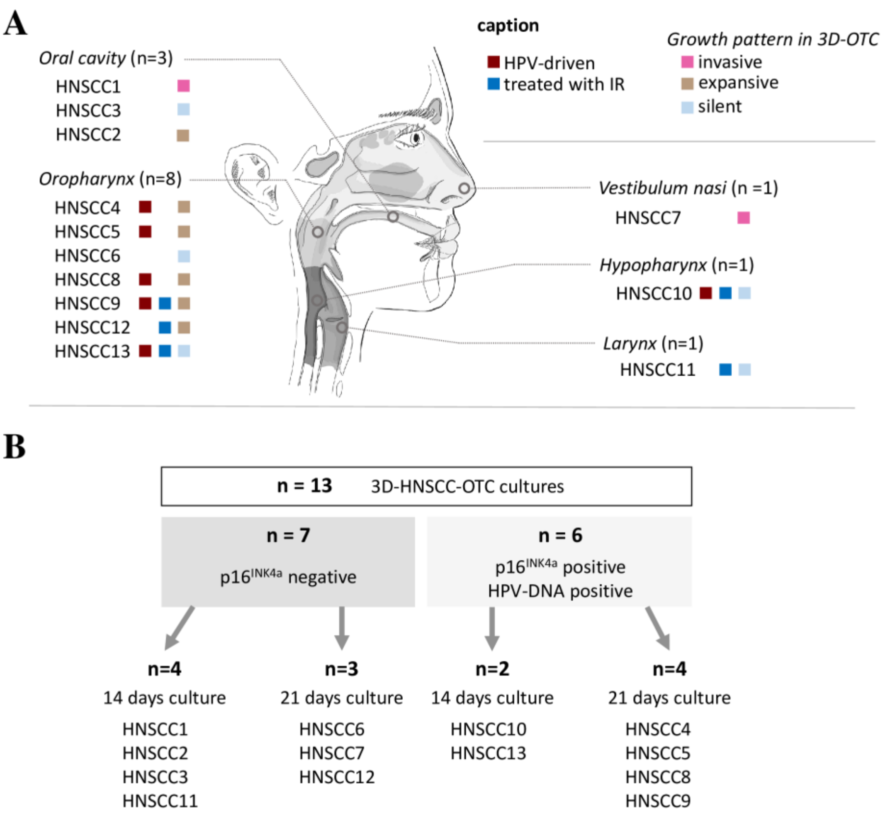

Figure S1. Specimen origin and experimental setting. **(A)** Depiction of specimen origin, HPV-status, treatment with fractionated IR and growth-pattern of 3D-OTC. **(B)** Details of HPV-status and time in culture of 3D-OTC. Abbreviations: IR, irradiation; HPV, human papillomavirus; 3D-OTC, 3D organotypic co-culture.

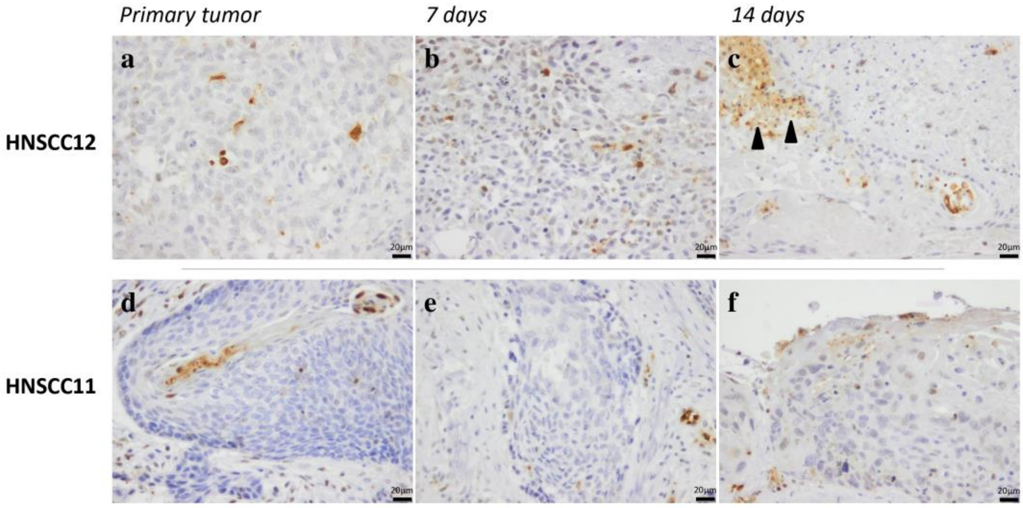

Figure S2. Visualization of apoptosis at different time points. Representative images of immunohistochemistry with an anti-cleaved caspase-3 antibody of two different HPV non-driven 3D-OTC cultures (*b-c* and *e-f*) and matching primary tumors (*a* and *d*) for visualization of apoptosis. HNSCC12 shows low levels of apoptotic cells in the primary with a slight increase during cultivation (see black arrowheads). HNSCC11 constantly low expression of cleaved caspase-3 presents in the

primary tumor and in 3D-OTC cultures. Abbreviations: HPV, human papillomavirus; 3D-OTC, 3D organotypic co-culture.

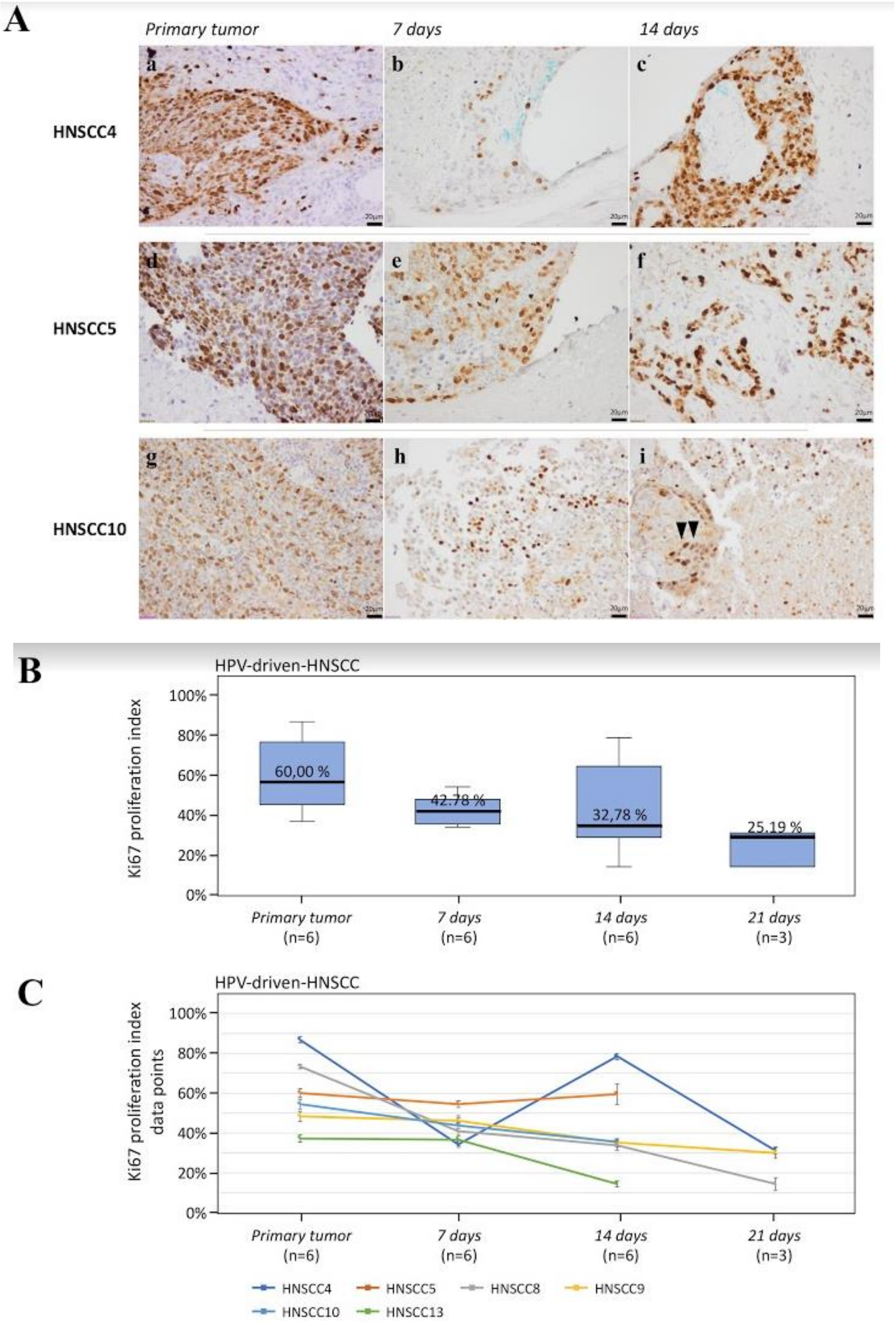

Figure S3. (A) IHC with an anti-ki-67-antibody of three different HPV-driven 3D-HNSCC-OTC (*b-c*; *e-f*; *h-i*) for the indicated time points and according primaries (*a*, *d*, *g*). **(B)** Boxplot of ki-67 proliferation indices of primaries and 3D- OTCs on day 7, 14, and 21 of all HPV driven HNSCC. **(C)** Mean values of ki-67 proliferation indices of all primaries and 3D-OTCs on day 7, 14, and 21 of all HPV driven HNSCC. Error bars indicate standard errors of the mean. Abbreviations: IHC, immunohistochemistry; HNSCC, Head and neck squamous cell carcinoma; 3D-OTC, 3D organotypic co-culture.

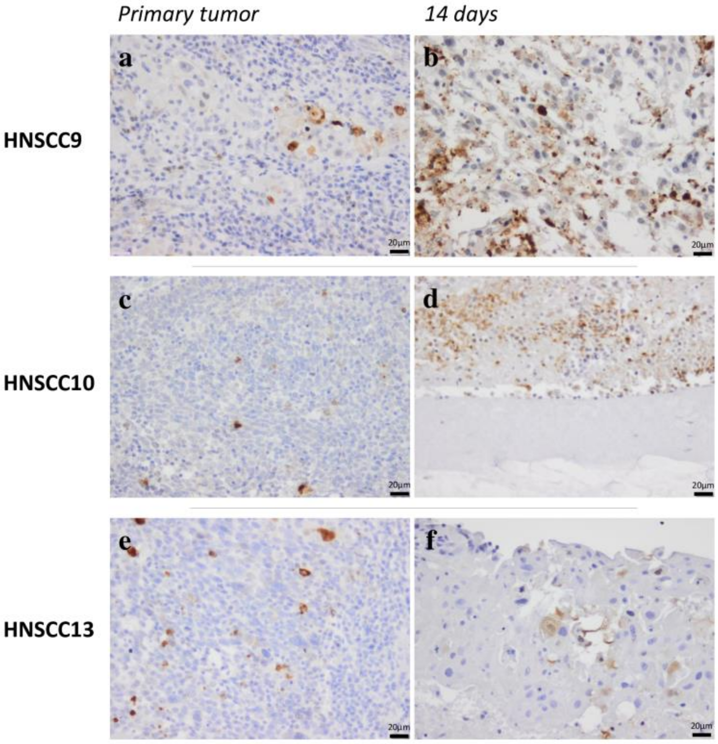

Figure S4. IHC with an anti-cc-3 antibody of three HPV-driven 3D-HNSCC-OTC on day 14 (*b, d, f*) and according primaries (*a, c, e*). HNSCC9 and HNSCC10 present an increase of cc-3 expression in 3D-HNSCC-OTC, compared to their primary. HNSCC13 maintains stable expression of cc-3 during culture. Abbreviations: IHC, immunohistochemistry; HPV, human papillomavirus; 3D-OTC, 3D organotypic co-culture; cc-3, cleaved caspase-3.

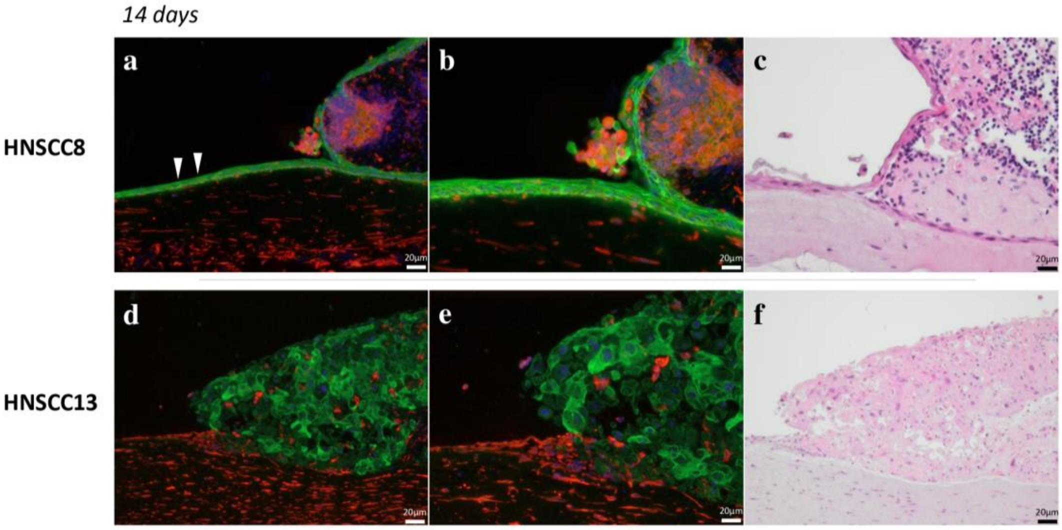

Figure S5. Co-Immunofluorescence staining with an anti-PanCK-antibody (green), an anti-vimentin-antibody (red) and DAPI (blue) (*a-b*; *d-e*) and H/E staining (*c, f*) of two different HPV-driven 3D-OTC on day 14. HNSCC8 presents an expansive growth pattern, white arrowheads (a) indicate the MF, HNSCC13 shows a silent growth pattern. Abbreviations: PanCK, pan-cytokeratin; H/E, Hematoxylin/eosin; HPV, human papillomavirus; 3D-OTC, 3D organotypic co-culture; MF, migration front.

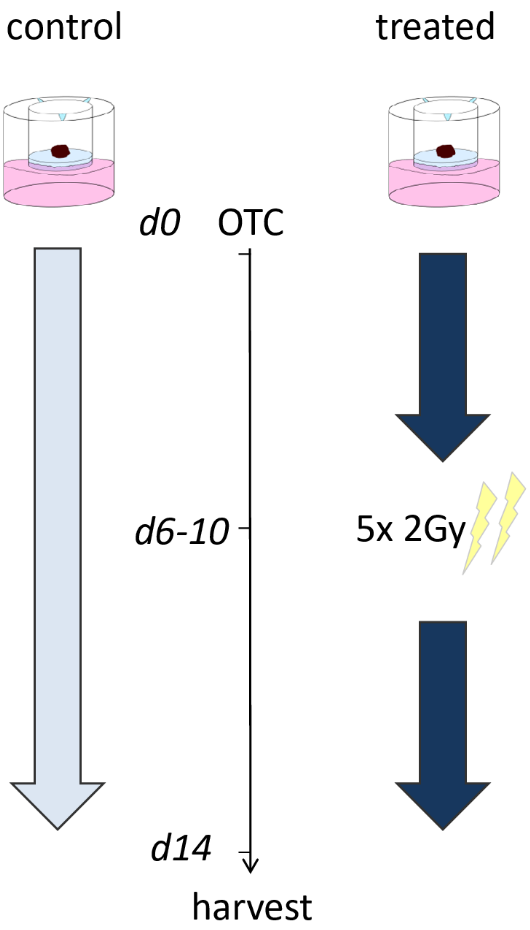

Figure 6. Diagram showing treatment with fractionated IR. After 5 days in culture 5 HNSCC-OTC were irradiated with 2 Gy on 5 consecutive days followed by 3 holidays before being harvested on day 14. Controls were mock-treated. Abbreviations: IR, irradiation; HNSCC, Head and neck squamous cell carcinoma; 3D-OTC, 3D organotypic co-culture.

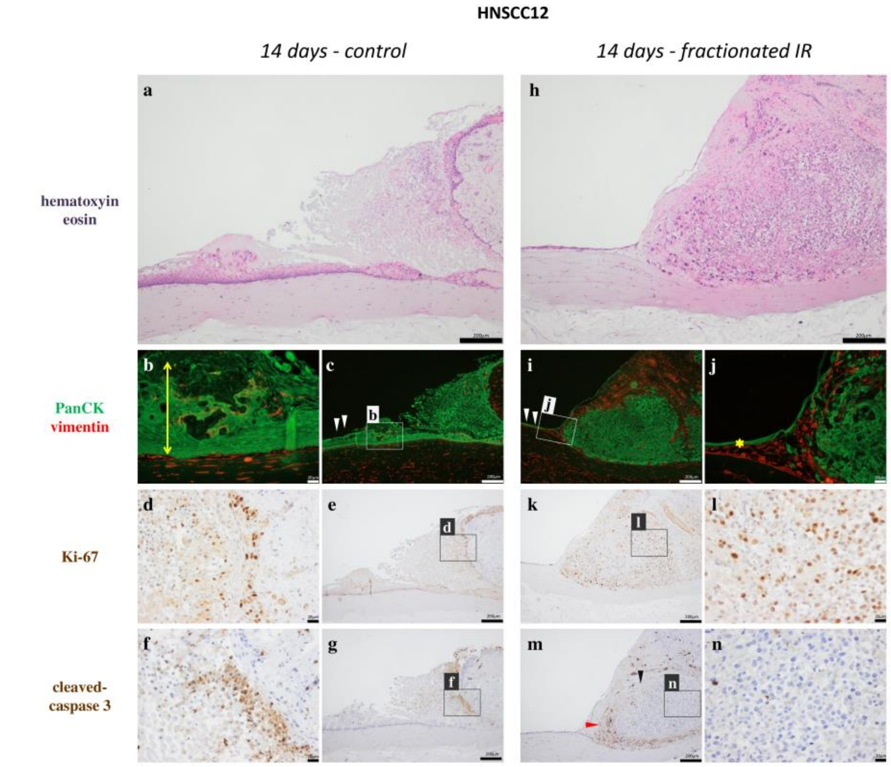

Figure 7. Selection of representative images of H/E, IF and IHC with indicated antibodies of one 3D-OTC, which was cultured for 14 days and treated with mock irradiation (*a-g*) or a fractionated irradiation scheme (*h-n*), in order to depict radiogenic impact. PanCK-vimentin-Co-IF reveals a reduction of layering in the migration front after fractionated irradiation (*see yellow flashs*) in comparision to the untreated sample. ki-67-IHC detects stable postradiogenic proliferation and IHC while cleaved caspase-3 shows heterogenous expression in the mock-treated and fractionated irradiated OTC. Apoptotic tumour cells (*see red arrowhead*) and those, not undergoing apoptosis (*see black arrowhead*). Abrreviations: H/E, haematoxylin/eosin; IF, immunofluorescence; IHC, immunohistochemistry; 3D-OTC, 3D organotypic co-culture; PanCK, pan-cytokeratin; IR, irradiation.

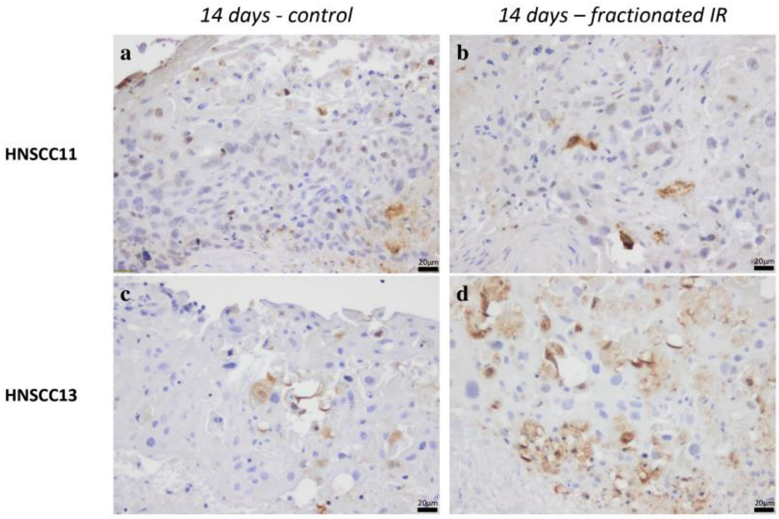

Figure S8. Impact of fractionated irradiation on apoptosis. Representative pictures of IHC with an anti-cc-3 antibody on 3D-OTC (all on day 14) of two different tumors; mock - (*a* and *c*) and matching fractionated-irradiated samples (*b* and, *d*), respectively. HNSCC11 shows similar intensity and

distribution of the cc-3 signal in the untreated OTC as well as after fractionated IR. Increasing cc-3 expression of fractional irradiated HNSCC13 compared to the mock-treated correlate. Abbreviations: IHC, immunohistochemistry; cc-3, cleaved caspase-3; 3D-OTC, 3D organotypic co-culture; IR, irradiation.

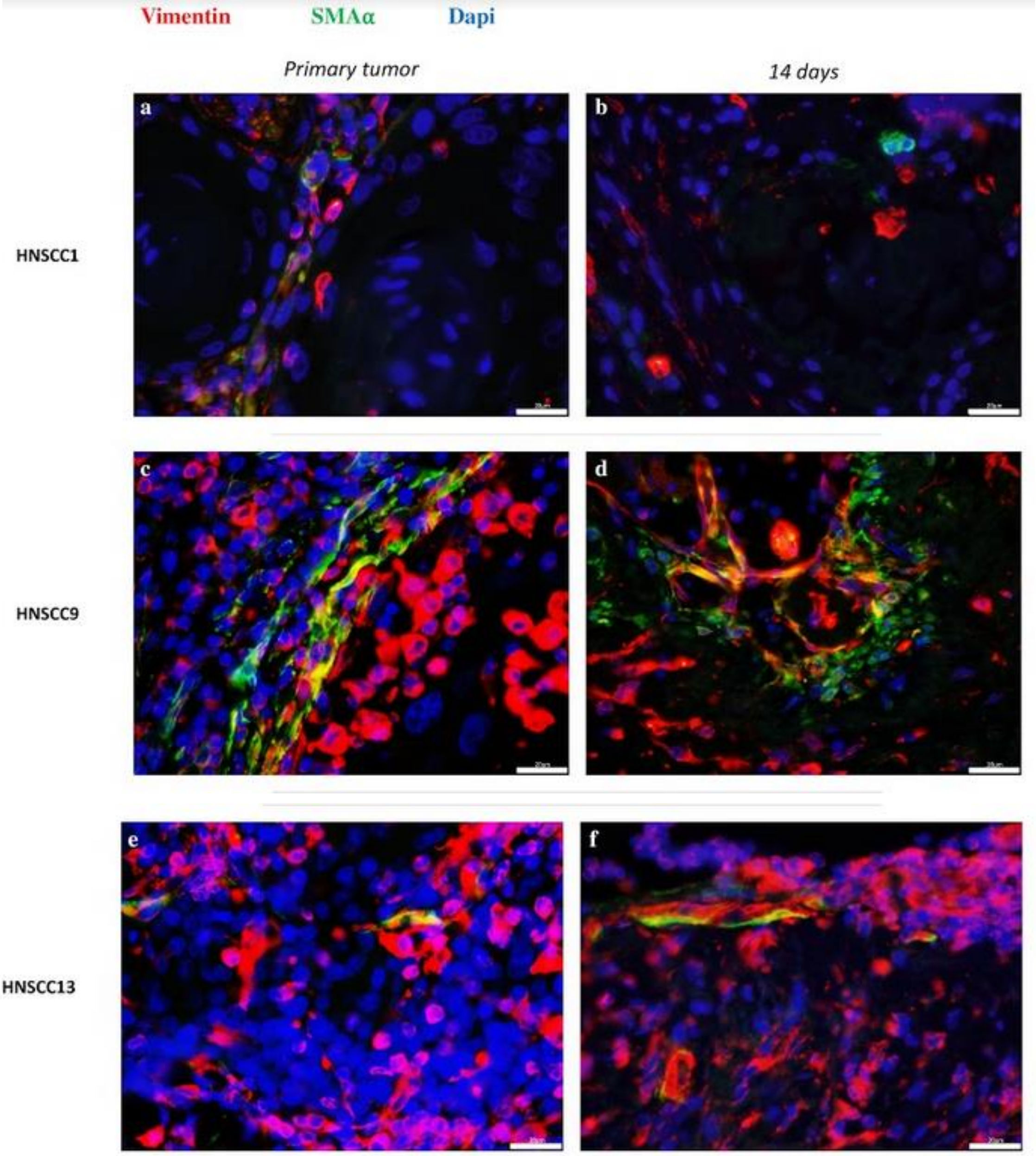

Figure S9. Representative pictures of co-IF staining for vimentin (red), ∝SMA (green) and DAPI (blue) of non-HPV-driven HNSCC1 (invasive type) and two HPV-driven HNSCC (HNSCC9; expansive type and HNSCC13; silent type in 3D-OTC on day 14 (*c, d, e*) and according primary tumor (*a, c, e*). Respective samples show a similar amount of ∝SMA positive cells in all primaries and according 3D-OTC on day 14. Abbreviations: IF, immunofluorescence; ∝SMA, ∝smooth muscle actin; HPV, human papillomavirus; HNSCC, Head and neck squamous cell carcinoma.
